# Optimization of synthetic human V_H_ affinity and solubility through in vitro affinity maturation and minimal camelization

**DOI:** 10.1002/pro.70114

**Published:** 2025-04-22

**Authors:** Kasandra Bélanger, Cunle Wu, Traian Sulea, Henk van Faassen, Deborah Callaghan, Annie Aubry, Marc Sasseville, Greg Hussack, Jamshid Tanha

**Affiliations:** ^1^ Human Health Therapeutics Research Centre, Life Sciences Division National Research Council Canada Ottawa Ontario Canada; ^2^ Medical Devices Research Centre, Life Sciences Division National Research Council Canada Montréal Quebec Canada; ^3^ Human Health Therapeutics Research Centre, Life Sciences Division National Research Council Canada Montréal Quebec Canada; ^4^ Department of Biochemistry, Microbiology and Immunology, Faculty of Medicine University of Ottawa Ottawa Ontario Canada

**Keywords:** affinity maturation, aggregation, camelization, human V_H_, single‐domain antibody, solubility, synthetic phage display library, V_H_H

## Abstract

An attractive feature of human V_H_s over camelid V_H_Hs as immunotherapeutics is their perceived lower risk of immunogenicity. While human V_H_s can readily be obtained from synthetic phage display libraries, they often suffer from low affinity and poor solubility compared to V_H_Hs derived from immune libraries. Using SARS‐CoV‐2 spike protein as a model antigen, we screened a synthetic human V_H_ phage display library and identified a diverse set of antigen‐specific V_H_s. However, the V_H_s exhibited low affinity, and many had low solubility; that is, they were prone to aggregation. To explore the feasibility of improving the affinity, we subjected a representative V_H_ to in vitro affinity maturation. We created a yeast surface display library of V_H_ variants employing a site‐saturated mutagenesis approach targeting complementarity‐determining regions and selected against the target antigen. Next‐generation sequencing of the selected variants, combined with structural modeling, identified a set of V_H_s as potentially improved candidates. Characterization of these candidates revealed several V_H_s with improved affinities of up to 100‐fold (*K*_D_s as low as 3 nM) and potent neutralization capabilities; however, they still showed significant aggregation. By introducing as few as two camelid residues into the framework region 2 of a high‐affinity V_H_ (a process referred to as camelization), we were able to completely solubilize the V_H_ without compromising its affinity and other important attributes, including thermostability and protein A binding. This study demonstrates the feasibility of generating high‐affinity, ‐solubility, and ‐stability human V_H_s from synthetic libraries through a combination of in vitro affinity maturation and minimal camelization.

## 1 INTRODUCTION

While traditional antibodies, for example, monoclonal antibodies (mAbs), are large heterotetrameric molecules composed of two heavy and two light chains, heavy‐chain antibodies from camelid species, for example, llamas, are devoid of light chain and the first heavy chain constant domain (C_H_1) (Hamers‐Casterman et al., 1993). Antigen recognition occurs through a single variable domain from the heavy chain, known as V_H_H or nanobody, which can be expressed autonomously maintaining the same intrinsic affinity as the parent full‐length antibody and displaying robust biophysical properties. As therapeutics, V_H_H single‐domain antibodies (sdAbs) offer several advantages and these have been extensively reviewed (Muyldermans, 2013; Steeland et al., 2016). Soluble, high‐affinity V_H_Hs are typically isolated through immunization of camelid species employing antibody discovery platforms such as phage display libraries. However, due to their camelid origin, these V_H_Hs usually need to be humanized to reduce their immunogenicity in humans (Fernandez‐Quintero et al., 2024; Rossotti, Belanger, et al., 2022). Although routine, this additional step in V_H_H therapeutic development can be resource‐intensive and, at times, may compromise the stability, solubility, and affinity of the V_H_Hs. The reliance on animal immunization for the generation of V_H_Hs raises production costs and extends development timelines. Depending on the nature and toxicity of the antigen, animal immune response can be unpredictable, and the large size of the animals being immunized is an added disadvantage (Belanger & Tanha, 2021).

Human antibody heavy chain variable domains (V_H_s) present an attractive alternative to camelid V_H_Hs due to their perceived lower risk of immunogenicity and the absence of the need for humanization. Fully immune human V_H_s that rival immune V_H_Hs in terms of affinity, stability, and solubility can be obtained from transgenic animals (Belanger & Tanha, 2021). However, these antibody discovery platforms remain dependent on immunization and are primarily accessible to biotech companies, limiting general lab use. Alternatively, rapid and efficient in vitro selection of human V_H_ sdAbs by combining high‐throughput phage display selection platforms with synthetic libraries has become a promising approach (Belanger & Tanha, 2021; Kim et al., 2014). In contrast to immune V_H_H/V_H_ approaches, synthetic libraries bypass immunization and library construction altogether. However, human V_H_s obtained from synthetic libraries often exhibit inadequate affinities and poor solubility, negatively impacting their safety, efficacy, and manufacturability. As a result, isolated synthetic human V_H_s often require further optimization through in vitro affinity maturation (Chan & Groves, 2021; Li et al., 2023) and solubilization engineering.

Mutagenesis‐based solubilization approaches for converting low solubility, aggregation‐prone human V_H_s into soluble, aggregation‐resistant domains have been comprehensively described (Kim et al., 2014). These approaches have involved introducing mutations in both the complementarity‐determining regions (CDRs) and framework regions (FRs). One of the earliest approaches, termed camelization, involved substituting FR2 hallmark positions 37, 44, 45, and 47 (Kabat numbering system (Kabat, 1991)) in the V_L_ (antibody light chain variable domain)/V_H_ interface of V_H_s with those typically found in V_H_Hs (Davies & Riechmann, 1994, 1995, 1996a, 1996b; Dottorini et al., 2004; Kim et al., 2014; Tanha et al., 2001) and thought to be responsible for the high solubility of V_H_Hs (Hamers‐Casterman et al., 1993; Kim et al., 2014). Such mutations have included V37F, G44E, L45R, and W47G/I, although in one instance a Y47 and not W47 was substituted (Tanha et al., 2001). The mutations are thought to improve the solubility of V_H_s by increasing the hydrophilicity of the V_L_/V_H_ interface (Kim et al., 2014). Camelization (V37F, G44E, L45R, W47G) has also been applied to rabbit and mouse V_H_s, which remained functional post‐camelization, although in both cases no direct evidence was presented to demonstrate the improved solubility of the V_H_s as a result of camelization (Aires da Silva et al., 2004; Pang et al., 2022).

Here, using a previously described synthetic V_H_ phage display library (Henry et al., 2017), we isolated a diverse set of severe acute respiratory syndrome coronavirus 2 (SARS‐CoV‐2) spike protein‐specific human V_H_s. As anticipated, the V_H_s had low affinities for their target antigen, in addition to being largely aggregating. We selected a lead V_H_ for in vitro affinity maturation, yielding variants with affinities improved by 50–100‐fold and potent in vitro virus neutralization capabilities. However, the affinity‐improved V_H_s were also aggregating, to a higher degree than the parent V_H_. Camelization of an affinity‐matured V_H_ at positions 44 and 45 (G44E/L45R) completely resolved its aggregation tendency without affecting its affinity and high thermostability. Adaptation of the strategy described here has the potential to provide a pipeline for the quick, reliable, and immunization‐independent generation of human V_H_s with high affinity, solubility, and stability from synthetic V_H_ libraries.

## 2 RESULTS

### 2.1 Identification and characterization of human V_H_s targeting the receptor‐binding domain of SARS‐CoV‐2 spike protein

**FIGURE 1:**
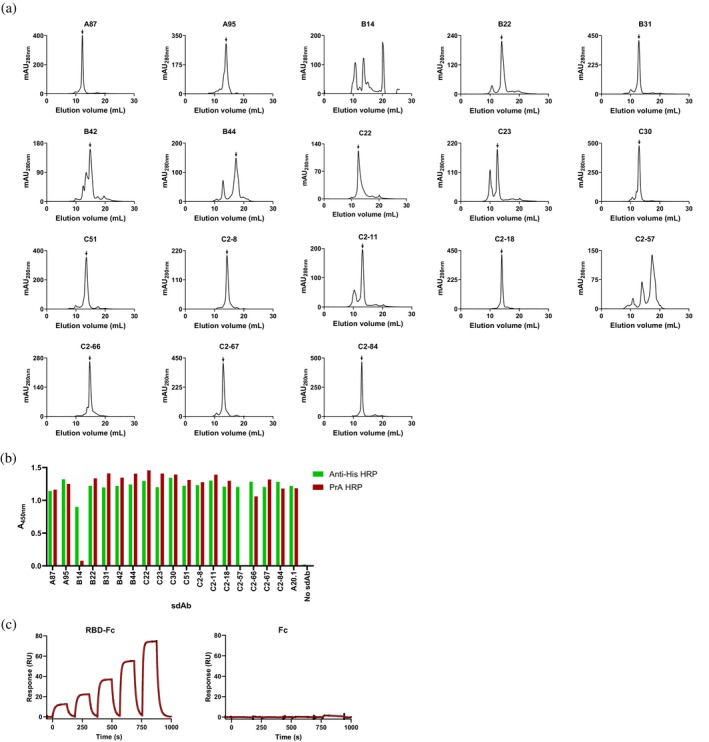
Characterization of V_H_s isolated by panning a human V_H_ phage‐display library against SARS‐CoV‐2 RBD. (a) SEC profiles showing the aggregation tendencies of human V_H_s. Monomeric peaks are marked by arrows, except for B14 and C2‐57, where the complexity of their SEC profiles precluded monomeric peak assignment. % monomer values calculated from the SEC profiles are reported in Table 1. mAU, milliabsorbance unit. (b) ELISA showing PrA binding to human V_H_s. Except for B14 and C2‐57, the remaining V_H_s and V_H_H A20.1 positive control (Hussack et al., 2011) bound to PrA and anti‐His_6_ tag antibody with similar strength, demonstrating the PrA binding property of human V_H_s. “No sdAb” represents assays in which V_H_ was omitted. (c) Representative SPR sensorgrams showing monomeric B22 binds in a concentration dependent manner to immobilized RBD‐Fc and not to the immobilized Fc, indicating the V_H_ is specific to RBD. Binding affinity and kinetic constants, determined by single‐cycle kinetic analysis using BIAevaluation 3.2 software, are reported in Table 1. Black lines represent raw data points; red lines are fits to a 1:1 binding model. SPR assays were performed in triplicates, and the results for one replicate is shown. RU, resonance unit.

In view of selecting sdAbs specific to the receptor‐binding domain (RBD) of the SARS‐CoV‐2 spike protein, a fully human V_H_ synthetic library (VHB82_SS_ (Henry et al., 2017)) was panned against three different forms of recombinant spike protein, including biotinylated RBD (Bio‐RBD), human IgG1 hinge/Fc‐fused RBD (RBD‐Fc) and full‐length spike protein, employing three different selection strategies (A, B, C; Figure S1). Following three rounds of panning, the presence of RBD‐specific clones was confirmed by polyclonal phage enzyme‐linked immunosorbent assays (ELISA) performed with amplified phage products from each round. The results demonstrated strong and specific binding to RBD, spike protein, and spike protein S1 subunit, with no binding to control antigens (Figure S2a). In the case of strategy C, where selection was performed with RBD‐Fc, binding to the human IgG1 hinge/Fc (Fc) was observed despite the fact that a negative selection step against the Fc was included during panning. Binding to the Fc‐lacking, His_6_‐tagged RBD‐H6, S1, and spike protein antigens confirmed the presence of RBD‐specific clones in the phage population. Eluted phage products from round 3 of panning were subcloned for expression in *Escherichia coli*. DNA sequencing of over 350 individual clones revealed the presence of 119 unique V_H_s, from which 18 showed specific binding to RBD‐Fc, with negligible or no binding to Fc or casein antigen controls, as shown by ELISA performed on V_H_‐containing bacterial lysates (Figure S2b). The sequences of these V_H_s are shown in Figure S3a. This initial pool of V_H_s was expressed at a larger scale and purified by immobilized metal‐ion affinity chromatography (IMAC). V_H_s were assessed for solubility by size‐exclusion chromatography (SEC; Figure 1a and Table 1). For the purpose of this study, we define solubility (interchangeably used with aggregation resistance) as the extent to which a V_H_ remains in its monomeric, native state. This is quantified as the percentage of monomer (% monomer). The majority of V_H_s (12) demonstrated low to no aggregation (% monomer 81%–98%) while six (B14, B42, B44, C23, C2‐11, and C2‐57) showed significant aggregation (% monomer <78%). Furthermore, with the exception of two (B14 and C2‐57), which were the most aggregating, the remaining 16 V_H_s were capable of protein A (PrA) binding in ELISA (Figure 1b and Table 1). Due to its poor expression, B14 was excluded from further analyses. Additional binding studies on the remaining 17 V_H_s, performed by surface plasmon resonance (SPR) assays with SEC‐purified monomeric V_H_ fractions against immobilized RBD‐Fc and Fc, revealed 10 V_H_s bound specifically to the RBD, albeit with low affinities, with B22 displaying the highest affinity of 480 nM (Figures 1c and S4, Table 1). The remaining seven V_H_s were either non‐binders (four V_H_s) or specific to the Fc (three V_H_s). B22 V_H_, which demonstrated the highest affinity, was chosen for affinity maturation experiments to improve its affinity.

**TABLE 1:** Biochemical properties of V_H_s selected from the VHB82_SS_ human V_H_ phage display library against SARS‐CoV‐2 RBD.

V_H_	% monomer	PrA binding	Binding specificity	*K*_D_ RBD (μM)^b^
B22	87	√	RBD	0.480 ± 0.09^c^
A95	84	√	RBD	3.97
B31	92	√	RBD	4.96^d^
B42	59	√	RBD	4.92^d^
C23	62	√	RBD	4.90^d^
C30	81	√	RBD	16.5^d^
C51	90	√	RBD	16.5^d^
C2‐11	78	√	RBD	6.73^d^
C2‐66	84	√	RBD	7.42^d^
C2‐67	94	√	RBD	18.8^d^
C22	95	√	Fc	–
C2‐8	96	√	Fc	–
C2‐18	98	√	Fc	–
A87	97	√	Non‐binder	–
B44	67	√	Non‐binder	–
C2‐84	98	√	Non‐binder	–
C2‐57^a^	nd	–	Non‐binder	–
B14^a^	nd	–	nd	nd

Abbreviation: nd, not determined.

^a^ The complexity of C2‐57 and B14 SEC profiles precluded determination of their % monomer values, and the scarcity of B14 due to poor expression did not allow characterization of B14 beyond PrA binding.

^b^ *K*_D_ for the binding of V_H_s to the RBD‐Fc. None of the RBD‐specific V_H_s bound to Fc (Figure S4).

^c^ Values are mean ± SD of three technical replicates. *k*a and *k*_d_ values were (1.25 ± 0.05) × 10^5^ M^−1^ s^−1^ and (5.99 ± 0.27) × 10^−2^ s^−1^, respectively.

^d^ Highest concentrations flowed were below the estimated *K*_D_s. Dash indicates no binding of V_H_ to PrA or RBD.

### 2.2 Affinity improvement of B22 V_H_ and in silico selection of lead candidates

Toward improving the affinity of B22, we created a yeast surface display (YSD) library of B22 variants employing a site saturation mutagenesis approach that targeted 37 positions in the three CDRs where a single codon or set of codons was substituted with all 20 amino acids at every position. The library cells were selected for surface expression and display of the B22 variants by probing for the presence of the C‐terminal c‐Myc‐tag by fluorescence‐activated cell sorting (FACS) of induced yeast cells. The cells were subsequently collected to serve as input (round 0, R0) for the first round of screening by FACS (Figure S5). Approximately 10^7^ cells were used for the first round of selection, which was performed against fluorescent‐labeled RBD‐Fc. Subsequent rounds of FACS selection were carried out with the monovalent RBD as opposed to the bivalent RBD‐Fc to minimize potential avidity effects and favor the selection of higher affinity V_H_ variants. Four rounds of selection were performed.

DNA of cells from round 3 selected for RBD binding (B22Lib‐R3) or unselected (B22Lib‐R0) were subjected to next‐generation sequencing (NGS) analysis. NGS data revealed that a single amino acid position (35) in CDR1 was highly enriched for Tyr, Trp, or Phe substitutions, five positions in CDR2 were moderately enriched for substitutions with Glu (position 51), Asn (position 52), Trp (position 57), Ser (position 58), and Thr (position 63), and a single position (100) in CDR3 was also moderately enriched for Asn substitution (Figure S6). Round 3 cells were sorted further to collect the top 5% of the round 4 binding population for DNA sequencing of purified colonies (total of 24 clones). All clones harbored the Tyr, Trp, or Phe substitutions at position 35, confirming the possible dominant contribution of these residues in improving the affinity of the V_H_ variants.

**FIGURE 2:**
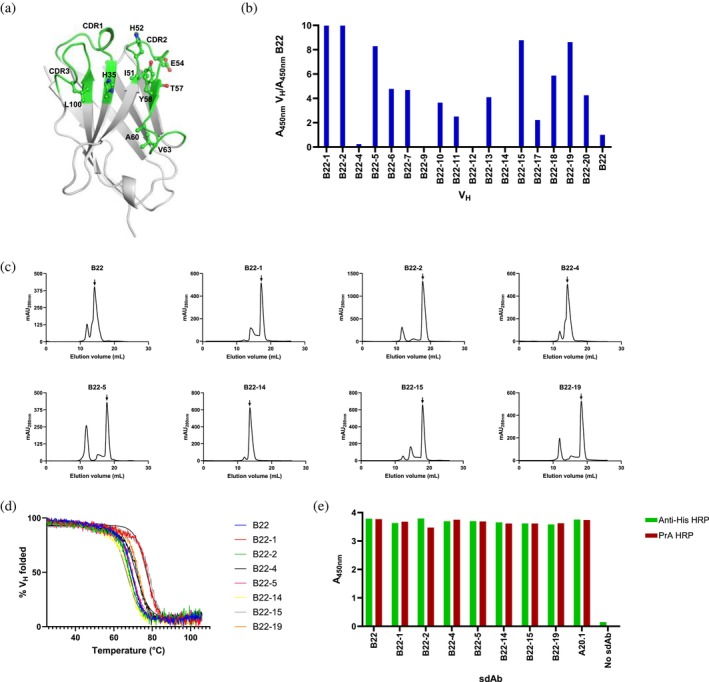
Affinity maturation of B22 V_H_ and preliminary binding and stability characterization. (a) Mapping of YSD‐based enriched mutations on the 3D model of the parent B22 V_H_. The model was built with the ABodyBuilder software. CDR loops are rendered in green. Positions where specific mutations led to improved antigen binding are shown in ball‐and‐stick models (side chains only) and labeled. The Kabat amino acid numbering is used (Kabat, 1991). For CDR designations, the Kabat/IMGT (CDR1), Kabat (CDR2) and IMGT (CDR3) systems are used (Kabat, 1991; Lefranc et al., 2003). (b) ELISA screening of B22 mutants for improved binding to RBD. The results are presented as the ratio of binding to RBD‐Fc of affinity‐matured V_H_s to B22 V_H_ (A_450nm_ V_H_/A_450nm_ B22). (c) SEC profiles showing the aggregation tendencies of V_H_s. Monomeric peaks are marked by arrows. % monomer values calculated from the graphs are reported in Table 3. mAU, milliabsorbance unit. (d) Thermal stability of V_H_s determined by CD experiments. V_H_ ellipticities measured as a function of temperature are normalized to percentage scales (% V_H_ folded). Melting temperatures (*T*_m_s), temperatures corresponding to 50% unfolded V_H_s, are reported in Table 3. Ellipticity values represent means of three technical replicates. (e) ELISA showing PrA binding by V_H_s. B22 parent and V_H_ variants as well as the A20.1 V_H_H positive control bound to HRP‐conjugated PrA (PrA HRP) and anti‐His tag antibody (anti‐His HRP) with similar strength, demonstrating the PrA binding property of the affinity‐matured V_H_s. “No sdAb” represents assays in which V_H_ was omitted.

The enriched substitutions were combined to generate double and triple mutants (Table 2). The CDR1 H35Y mutation was selected as the representative of the three dominant aromatic substitutions at position 35. Initially, all inter‐CDR double‐mutant combinations involving enriched mutations in CDR1 and CDR3 (variant B22‐1), CDR1 and CDR2 (variants B22‐2–B22‐6) or CDR2 and CDR3 (variants B22‐7–B22‐11) were designed. In order to reduce the number of possible intra‐CDR2 double‐mutant and inter‐CDR triple‐mutant combinations, enriched positions were mapped on a 3D structural model of the original B22 V_H_ (Figure 2a). Since the I51 and V63 residues in CDR2 were predicted to be solvent inaccessible, their substitutions were deprioritized. Hence, only the remaining three enriched substitutions in CDR2 were combined to design the final three intra‐CDR2 double mutants (variants B22‐12–B22‐14) and three inter‐CDR1/2/3 triple mutants (variants B22‐15–B22‐17). Three individual top‐binding clones consisting of double mutants were also selected for production and analysis (variants B22‐18–B22‐20). These clones contained mutations in CDR2 positions 51, 54, or 60 and the dominant H35Y mutation in CDR1.

**TABLE 2:** List of double and triple mutants for affinity‐matured B22 V_H_s.

Variant	Mutations		
	CDR loop(s)	CDR positions	Source
B22‐1	1, 3	H35Y, L100N	YSD DM enrichment
B22‐2	1, 2	H35Y, I51E	YSD DM enrichment
B22‐3	1, 2	H35Y, T57W	YSD DM enrichment
B22‐4	1, 2	H35Y, Y58S	YSD DM enrichment
B22‐5	1, 2	H35Y, V63T	YSD DM enrichment
B22‐6	1, 2	H35Y, H52N	YSD DM enrichment
B22‐7	2, 3	I51E, L100N	YSD DM enrichment
B22‐8	2, 3	T57W, L100N	YSD DM enrichment
B22‐9	2, 3	Y58S, L100N	YSD DM enrichment
B22‐10	2, 3	V63T, L100N	YSD DM enrichment
B22‐11	2, 3	H52N, L100N	YSD DM enrichment
B22‐12	2	T57W, Y58S	YSD DM enrichment and structural model
B22‐13	2	H52N, T57W	YSD DM enrichment and structural model
B22‐14	2	H52N, Y58S	YSD DM enrichment and structural model
B22‐15	1, 2, 3	H35Y, H52N, L100N	YSD DM enrichment and structural model
B22‐16	1, 2, 3	H35Y, T57W, L100N	YSD DM enrichment and structural model
B22‐17	1, 2, 3	H35Y, Y58S, L100N	YSD DM enrichment and structural model
B22‐18	1, 2	H35Y, I51S	YSD individual top‐binding clone
B22‐19	1, 2	H35Y, A60T	YSD individual top‐binding clone
B22‐20	1, 2	H35Y, E54R	YSD individual top‐binding clone

Abbreviations: DM, deep mutagenesis; YSD, yeast surface display.

Expression plasmids encoding the 20 potential affinity‐improved V_H_s (B22‐1–B22‐20) were synthesized (Figure S3b). Following in vitro expression and biotinylation, their ability to bind RBD relative to the parent B22 V_H_ was determined by ELISA. To this end, biotinylated‐V_H_‐containing samples were captured on streptavidin‐coated wells, followed by incubation with RBD‐Fc and detection with an anti‐Fc antibody. Probing of the captured V_H_s by ELISA against His_6_ tag revealed B22‐3, B22‐8, and B22‐16 were not expressed in sufficient quantities and were excluded from further analyses. Results for the remaining V_H_s showed many had improved binding of ~2–10‐fold (Figure 2b), while four (B22‐4, B22‐9, B22‐12, B22‐14) had reduced binding when compared to B22. In particular, B22‐1, B22‐2, B22‐5, B22‐15, and B22‐19, which showed similar captured V_H_s on ELISA microtiter wells, showed improved binding of ~8–10‐fold. These five B22 variants were selected for further validation and characterization. B22‐4 and B22‐14, which showed reduced binding, were included as controls.

### 2.3 Stability characterization of affinity‐matured B22 variants

Following bacterial expression of seven B22 variants and parent B22 and their subsequent purification by IMAC, the V_H_s were assessed by SEC for aggregation propensity (Figure 2c and Table 3). With the exception of B22‐14, which was essentially aggregation free (% monomer 97%), and B22‐4, which was highly monomeric (% monomer 91%), the remaining mutant V_H_s displayed varying degrees of aggregation (% monomer 49%–79%) which were higher than that of the parent B22 V_H_ (% monomer 87%), with B22‐5 showing the highest degree of aggregation (% monomer 49%). The monomer‐peak elution volumes (*V*_e_s) of the aggregating mutants increased from 14.0 mL (B22) to 17.2–18.4 mL, whereas those of the less/non‐aggregating B22‐4 and B22‐14 variants remained largely unchanged from B22 (*V*_e_ 14.2 mL and 13.6 mL, respectively). This suggests non‐specific interactions of the aggregating mutants with the SEC column, a behavior consistent with their aggregation tendencies. The possibility that the larger‐than‐expected *V*_e_s of the aggregating mutants could be due to smaller fragment V_H_ derivatives is ruled out by mass spectrometry, which confirmed that the mutant V_H_s were intact (data not shown). Interestingly, the non‐aggregating B22‐14 had the highest expression yield of all V_H_s.

**TABLE 3:** Physico‐chemical and functional properties of affinity‐improved B22 V_H_s.

V_H_	Expression yield (mg/L)^a^	% monomer	*V*_e_ M (mL)^b^	*T*_m_ (°C)^c^	PrA binding	*EC*_50_ (nM) (ELISA)^c^	*K*_*D*_ (nM) (SPR)^c^, ^d^	*EC*_50_ (nM) (CCA)^c^, ^e^	*IC*_50_ (nM)^c^
B22	24	87	14.0	68.9 ± 1.0	√	–	295 ± 1	–	–
B22‐1	30.3	65	17.2	77.1 ± 0.7	√	1.8 ± 0.5	3.56 ± 0.03	nd	nd
B22‐2	8.7	79	17.7	67.8 ± 0.5	√	2.7 ± 0.4	8.30 ± 0.04	nd	nd
B22‐4	22.4	91	14.2	71.4 ± 1.0	√	–	490 ± 4	nd	nd
B22‐5	18.2	49	17.8	69.2 ± 0.8	√	2.1 ± 0.5	7.61 ± 0.01	nd	nd
B22‐14	33.3	97	13.6	66.6 ± 0.5	√	–	7257 ± 475	nd	nd
B22‐15	23.2	63	18.1	77.8 ± 1.2	√	1.5 ± 0.2	3.08 ± 0.02	3.8 ± 1.3	29.8 ± 7.4
B22‐19	14.1	73	18.4	72.6 ± 0.8	√	2.8 ± 0.9	8.47 ± 0.02	1.7 ± 0.1	22.8 ± 8.3

Abbreviation: nd, not determined.

^a^ Expression yield is expressed in terms of mg of purified protein per liter of bacterial culture.

^b^ Elution volume of the monomeric peak (*V*_e_ M) obtained from size‐exclusion chromatograms.

^c^ Values are mean ± SD of two to three technical replicates.

^d^ *K*_D_s are for the binding of V_H_s to RBD‐Fc. None of the V_H_s bound to Fc or ovalbumin (Figures S7 and S8). The highest concentration of B22‐14 flowed was below the estimated *K*_D_. *k*_a_ and *k*_d_ values are reported in Table S1.

^e^ *EC*_50_s were determined by cell cytometry assays (CCA) of V_H_s against CHO‐Spike cells. Dash indicates no binding or neutralization observed.

Further assessment of the affinity‐matured V_H_ variant stability was carried out by determining their melting temperatures (*T*_m_s) in circular dichroism (CD) assays. All V_H_s were thermostable with a *T*_m_ range and median of 66.6–77.8°C and 70.3°C, respectively (Figure 2d and Table 3). Compared to B22 V_H_, the mutants had either the same (B22‐5), lower by a few degrees (B22‐2, B22‐14) or higher (B22‐1, B22‐4, B22‐15, B22‐19) *T*_m_s. The aggregation‐free B22‐14 had the lowest *T*_m_ of 66.6°C. Finally, all V_H_s bound to the V_H_ conformational probe PrA as determined by ELISA (Figure 2e and Table 3), indicating they maintained their immunoglobulin (Ig) fold with mutation.

### 2.4 Binding characterization of affinity‐matured B22 variants

**FIGURE 3:**
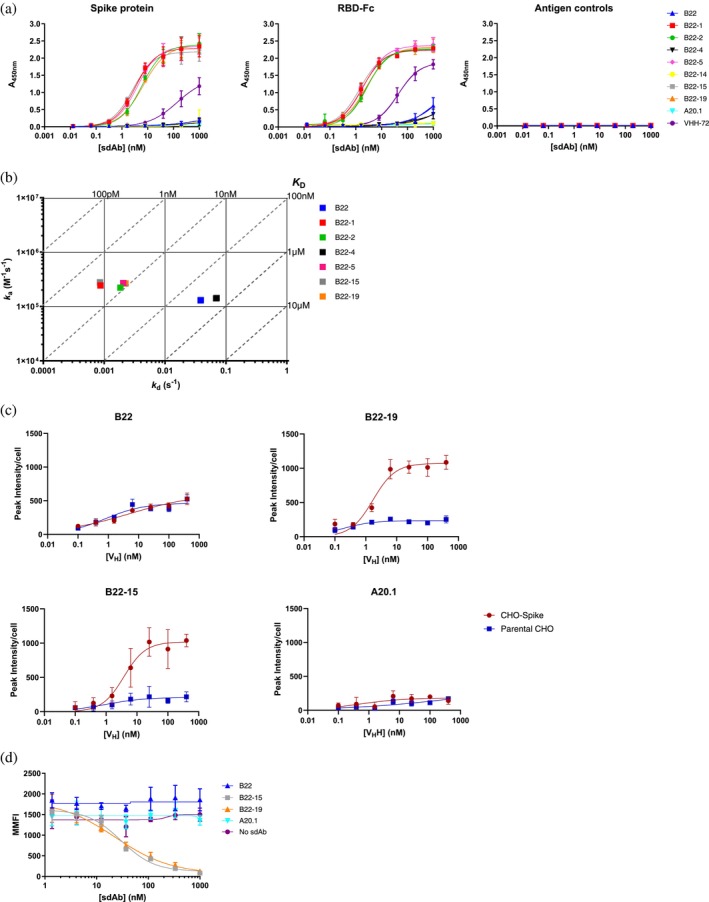
Binding and neutralization potency of affinity‐matured V_H_s. (a) ELISA showing the improved affinity and specificity of the mutant B22 V_H_s B22‐1, B22‐2, B22‐5, B22‐15 and B22‐19. VHH‐72 V_H_H (Wrapp et al., 2020a; Wrapp et al., 2020b) and *Clostridioides difficile* toxin A‐specific A20.1 V_H_H (Hussack et al., 2011) are positive and negative antibody controls, respectively. ‘Antigen controls’ includes Fc, human serum albumin and casein. Error bars indicate standard deviations of means of three technical replicates. *EC*_50_ affinity constants obtained from “RBD‐Fc graph” (middle panel) are reported in Table 3. (b) On−/off‐rate maps summarizing kinetic rate constants (*k*_a_s and *k*_d_s) of affinity‐matured V_H_s for binding to RBD‐Fc as determined by SPR. B22‐14 V_H_ is excluded due to the absence of rate constant data. Diagonal lines represent equilibrium dissociation constants, *K*_D_s. None of the V_H_s bound to control Fc (Figure S7) or ovalbumin (Figure S8). Each data point is the mean of three technical replicates. *k*_a_s/*k*_d_s and *K*_D_s are reported in Tables S1 and 3. (c) Cell cytometry assay showing the improved bindings and specificity of two lead affinity‐matured V_H_s, B22‐15, and B22‐19, to CHO‐Spike cells expressing spike protein on their surface. Error bars indicate standard deviations of means of two to three technical replicates. *EC*_50_s obtained from graphs are reported in Table 3. (d) Surrogate virus neutralization assay demonstrating the potency of B22‐15 and B22‐19 V_H_s in inhibiting the binding of RBD‐Fc to HEK293T cells expressing the ACE2 receptor. “No sdAb” represents assays in which V_H_ was omitted. A20.1 V_H_H (Hussack et al., 2011) and “no sdAb”, which do not show any detectible inhibitory activity, are negative antibody controls. Error bars indicate standard deviations of means of three technical replicates. *IC*_50_s for B22‐15 and B22‐19 are reported in Table 3. MMFI, median mean fluorescence intensity.

The binding of purified V_H_ variants to SARS‐CoV‐2 RBD‐Fc and spike protein was assessed by ELISA and SPR. Consistent with the results obtained in the preliminary binding assays (Figure 2b), by ELISA, B22‐1, B22‐2, B22‐5, B22‐15, and B22‐19 showed improved affinities (i.e., decreased half‐maximal effective concentrations [*EC*_50_s] of 1.5–2.8 nM) and B22‐4 and B22‐14 demonstrated reduced affinities compared to the parent B22 V_H_ (Figure 3a and Table 3). Binding was specific as none of the V_H_s bound Fc, human serum albumin, or casein. Additionally, negative control A20.1 V_H_H (Hussack et al., 2011) did not interact with any of the antigens, and the positive control VHH‐72 V_H_H (Wrapp et al., 2020a, 2020b) bound RBD and spike protein, albeit unsurprisingly weakly. These results were further corroborated in subsequent SPR experiments performed with V_H_s against RBD‐Fc, Fc, as well as ovalbumin, a polyspecificity reagent known to be effective for detecting antibody non‐specific interactions (Makowski et al., 2021) (Figures 3b, S7, and S8, Table 3). While compared to the parent B22 V_H_ (*K*_D_ 295 nM), B22‐4 and B22‐14 showed lower affinities (higher *K*_D_s) of 490 and 7257 nM, respectively, the remaining five affinity‐improved V_H_s displayed much higher affinities (*K*_D_s of 3.08–8.47 nM). None of the V_H_s bound to the Fc or ovalbumin, confirming that the V_H_s were specifically directed to the RBD. Notably, V_H_s B22‐1 and B22‐15 showed a ~ 100‐fold affinity improvement compared to the parent B22 V_H_ (Figures 3b and S7, Table 3). The affinity improvements were largely due to decreases (improvements) in dissociation rate constants, *k*_d_s, rather than increases in association rate constants, *k*_a_s (Table S1). The H35Y substitution was observed in all five affinity‐improved V_H_s, confirming the beneficial effect of this mutation on affinity as suggested by the YSD/NGS data (Figure S3 and Table 2). Consistent with this, three of the four V_H_s with diminished affinities (B22‐4, B22‐9, B22‐12, B22‐14) lacked the 35Y substitution (Figure 2b and Table 2). We observed that the five affinity‐improved V_H_s (B22‐1, B22‐2, B22‐5, B22‐15 and B22‐19) had high *V*_e_s of 17.2–18.4 mL, while the two non‐improved V_H_s, B22‐4 and B22‐14, had much lower *V*_e_s of 14.2 and 13.6 mL, respectively, suggesting subtle non‐specific interactions may contribute to the improved affinities of the former five V_H_s. Affinity‐matured V_H_s fell into two similar affinity clusters: B22‐15/B22‐1 cluster with *K*_D_s of 3.08–3.56 nM, and B22‐5/B22‐2/B22‐19 cluster with *K*_D_s of 7.61–8.47 nM (Figure 3b and Table 3). B22‐15 and B22‐19 V_H_s, each representing one of the two clusters, were chosen for further analysis.

Next, the ability of the B22‐15 and B22‐19 variants to interact with the spike protein in a more natural context, that is, in the cell membrane, was determined by cell cytometry using Chinese hamster ovary (CHO) cells displaying spike proteins on their surface (CHO‐Spike). B22 showed a similar, concentration‐dependent binding to both CHO‐Spike and CHO (spike protein negative) cells, which was higher than the binding observed with a negative control V_H_H against the same cells, indicating some degree of non‐specific antibody–antigen interactions for B22 (Figure 3c and Table 3). In contrast, and consistent with the ELISA and SPR results, the affinity‐improved B22‐15 and B22‐19 V_H_s demonstrated significantly increased binding to CHO‐Spike cells compared to B22 V_H_, with *EC*_50_ values of 3.8 and 1.7 nM, respectively (Figure 3c and Table 3). The binding of both V_H_s to the parent CHO cells was at background levels comparable to that seen for the negative control V_H_H, indicating that the binding of the V_H_s to CHO‐Spike was specific to the spike protein.

### 2.5 Assessing the neutralization potencies of affinity‐improved B22 variants

To determine whether the improved affinities of B22‐15 and B22‐19 V_H_s translated into more potent virus neutralization, a cell cytometry‐based surrogate neutralization assay was performed. The assay measured the ability of the V_H_s to block the interaction of SARS‐CoV‐2 RBD (RBD‐Fc) with the human angiotensin‐converting enzyme 2 (ACE2) receptor that is expressed on the surface of human embryonic kidney cells (HEK293T ACE2) in terms of half‐maximal inhibitory concentrations (*IC*_50_s). As shown in Figure 3d and Table 3, and consistent with earlier binding data, both B22‐15 and B22‐19 were able to effectively inhibit the RBD‐Fc/ACE2 interaction (*IC*_50_s of 29.8 and 22.8 nM, respectively) in contrast to the parent B22 V_H_, which did not show any inhibitory activity.

### 2.6 Camelization of B22‐15

**FIGURE 4:**
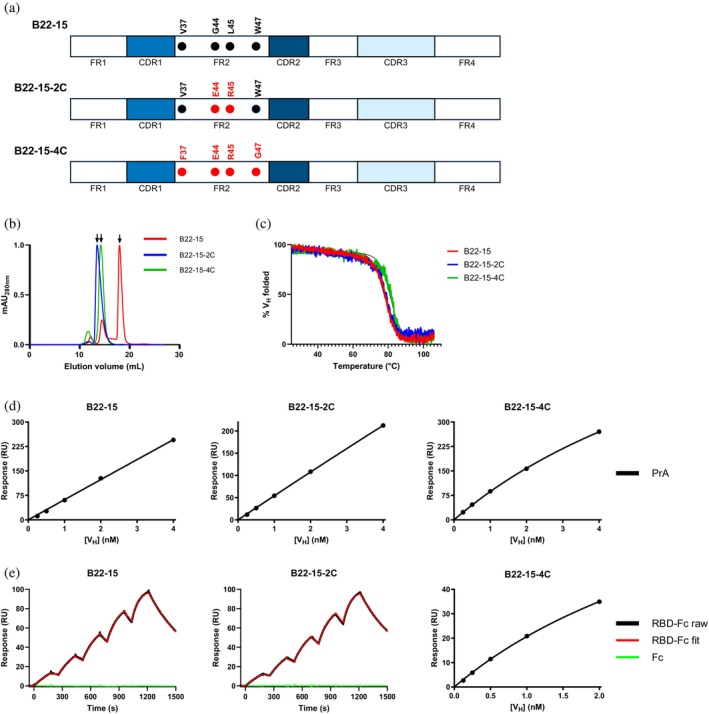
Camelization of B22‐15 V_H_. (a) Schematic diagram comparing camelized B22‐15‐2C and B22‐15‐4C V_H_s to B22‐15 V_H_ with mutated FR2 positions 37, 44, 45, and 47 (Kabat numbering system (Kabat, 1991)) shown. B22‐15‐2C and B22‐15‐4C differ from B22‐15 at two and four positions, respectively. CDR, complementarity‐determining region; FR, framework region. (b) SEC profiles comparing the aggregation tendencies of B22‐15, B22‐15‐2C, and B22‐15‐4C V_H_s. Monomeric peaks are marked by arrows. % monomer values and monomer‐peak elution volumes are reported in Table 4. mAU, milliabsorbance unit. (c) Melting curve profiles comparing the thermal stability of B22‐15, B22‐15‐2C, and B22‐15‐4C V_H_s determined by CD experiments. V_H_ ellipticities measured as a function of temperature are normalized to percentage scales (% V_H_ folded). Ellipticity values represent means of three technical replicates. Melting temperature (*T*_m_s), temperatures corresponding to 50% unfolded V_H_s, are reported in Table 4. (d, e) Representative SPR sensorgrams and steady‐state plots comparing the binding of human and camelized B22‐15 V_H_s to PrA (d) and RBD‐Fc target antigen (e). None of the V_H_s bound to negative antigen control Fc (green lines; data not shown for B22‐15‐4C) or ovalbumin (Figure S8). *k*_a_s/*k*_d_s and *K*_D_s for the binding of V_H_s to RBD‐Fc are reported in Tables S2 and 4. SPR assays were performed in triplicates, and the results for one replicate is shown as representative profile. Black lines (RBD‐Fc raw) represent raw data points; red lines (RBD‐Fc fit) are fits to a 1:1 binding model. RU, resonance unit.

Between B22‐15 and B22‐19 V_H_s, B22‐15 showed the higher antigen binding affinity by SPR; however, it aggregated considerably. We hypothesized that targeted camelization, involving substituting residues at V_H_ positions 37, 44, 45, and 47 with those typically found in camelid V_H_Hs and shown to improve aggregation resistance of V_H_s, would solubilize B22‐15 without adversely impacting stability and binding. We made two camelized versions of B22‐15 V_H_: (i) a minimally‐camelized V_H_ (B22‐15‐2C) with the G44E/L45R mutation set, (ii) and a camelized V_H_ (B22‐15‐4C) with the V37F/G44E/L45R/W47G mutation set (Figure 4a). SEC analysis revealed a stepwise improvement in B22‐15 solubility with the decreasing levels of camelization (Figure 4b and Table 4). First, the % monomer of B22‐15 (63%) was improved to 88% for B22‐15‐4C and to 97% for B22‐15‐2C. Second, consistent with the % monomer data, monomer *V*_e_s were decreased from 18.1 mL for B22‐15 to 14.2 mL for B22‐15‐4C and further to 13.4 mL for B22‐15‐2C. These experimental results were independently validated through in silico predictions of solubility based on modeled 3D structures. The predicted solubility trends for B22‐15, B22‐15‐2C, and B22‐15‐4C aligned with the solubility trends observed in the SEC experiments (Figure S9). Altogether, these data demonstrated that as little as two camelizing mutations (G44E/L45R) were sufficient to convert an aggregating human V_H_ to a non‐aggregating one.

**TABLE 4:** Physico‐chemical properties of camelized B22‐15 V_H_s.

V_H_	% monomer	*V*_e_ M (mL)^a^	*T*_m_ (°C)^b^	*K*_D_ PrA (μM)^b^, ^c^	*K*_D_ RBD (nM)^b^, ^d^
B22‐15	63	18.1	77.9 ± 1.2	32.5 ± 0.6	5.96 ± 0.04
B22‐15‐2C	97	13.4	78.1 ± 0.9	62.6 ± 6.1	6.49 ± 0.05
B22‐15‐4C	88	14.2	81.2 ± 0.0	10.3 ± 0.1	3880 ± 100

^a^ Elution volume of the monomeric peak (*V*_e_ M) obtained from size‐exclusion chromatograms.

^b^ Values are mean ± SD of two or three technical replicates.

^c^ *K*_D_ PrA denotes the *K*_D_ obtained by SPR assays for the binding of V_H_s to immobilized PrA. Highest concentrations of V_H_s flowed were below the estimated *K*_D_s.

^d^ *K*_D_ RBD denotes the *K*_D_ obtained by SPR assays for the binding of V_H_s to immobilized RBD‐Fc. V_H_s did not bind to immobilized Fc (Figures 4 and S4) or ovalbumin (Figure S8). *k*_a_ and *k*_d_ values are reported in Table S2.

In terms of thermostability, camelized B22‐15‐2C V_H_ had a *T*_m_ of 78.1°C, slightly lower than that for camelized B22‐15‐4C (81.2°C) but similar to that for B22‐15 (77.9°C) (Figure 4c and Table 4). Camelization did not eliminate B22‐15 PrA binding, as determined by SPR (Figure 4d and Table 4). Similar *K*_D_s of 32.5 μM and 62.6 μM were observed for B22‐15 and B22‐15‐2C, respectively (Table 4). However, B22‐15‐4C had a *K*_D_ of 10.3 μM, ~3‐ and ~6‐fold lower than that of B22‐15 and B22‐15‐2C, respectively. It should be noted that the highest concentrations of V_H_s used in the SPR assays were below the estimated *K*_D_s. Therefore, these *K*_D_s should be interpreted with caution, as estimates only. Finally, the RBD binding affinity of B22‐15‐2C was essentially the same as that of B22‐15 (*K*_D_ 6.49 nM and 5.96 nM, respectively); however, that of B22‐15‐4C was drastically lower, by ~700‐fold (*K*_D_ 3880 nM; Figure 4e, Tables 4 and S2). Neither B22‐15‐2C nor B22‐15‐4C bound to Fc or ovalbumin, indicating that the camelized V_H_s were specifically directed to the RBD (Figures 3e and S8).

## 3 DISCUSSION

Two key features of camelid V_H_Hs and human V_H_s derived from animal immunization are high affinity and solubility. Here, we demonstrate that by combining in vitro affinity maturation with camelization, antigen‐specific human V_H_s can be derived from synthetic phage display libraries, which, like immune‐derived V_H_Hs and V_H_s, exhibit both high solubility and high affinity. However, unlike immune V_H_Hs and V_H_s, they avoid the drawbacks associated with animal immunization (Belanger & Tanha, 2021).

The VHB82_SS_ synthetic V_H_ library used in this study was constructed on a scaffold selected for its PrA binding property, ultrahigh stability, and tolerance to CDR randomization (Henry et al., 2017). This approach was designed to maximize library diversity while preserving favorable biophysical properties of the V_H_s. However, it was noted that some CDR‐shuffled clones exhibited poor stability and solubility, which could not be entirely eliminated and might emerge during selection against certain antigens. It was therefore not surprising that several of the 18 V_H_s isolated from panning the VHB82_SS_ library against SARS‐CoV‐2 RBD were aggregation prone. Given the non‐immune, synthetic nature of the library, it was also unsurprising that, with the exception of B22, the affinities of RBD‐specific V_H_s were low, in the micromolar *K*_D_ range (3.97–18.8 μM). While B22 showed relatively higher affinity than others by at least ~10‐fold, this was not in a therapeutically useful range typically seen for immune sdAbs and required further affinity improvement. We currently lack a clear understanding of the factors influencing the number of specific binders isolated, their affinities, and biophysical properties. As suggested previously (Henry et al., 2017), the recovery of rare sdAb specificities may depend on the library itself, the target antigen's quality, size or density, or the selection methodology. Importantly, the isolated V_H_s retained the PrA binding characteristic of the parent library scaffold, despite significant deviations in CDR sequence and length; only two V_H_s, B14 and C2‐57, which were highly aggregating and likely misfolded, did not bind PrA, consistent with PrA's selective affinity for folded domains (Bond et al., 2003; Chang et al., 2014; Julian et al., 2015). This property is advantageous, allowing for the purification of tag‐less V_H_s via PrA affinity chromatography, a standard method in the industrial purification of therapeutic antibodies (Sara Kanje et al., 2020; Shi & Sun, 2021).

In vitro affinity maturation experiments indicated that as few as two CDR mutations could enhance V_H_ affinity by up to ~100‐fold, primarily through improved off‐rates (*k*_d_s). The CDR1 H35Y mutation was notably dominant in enhancing affinity. In addition to this beneficial H35Y mutation, both affinity‐enhanced B22‐1 and B22‐15 also carried the L100N mutation in CDR3, suggesting that this mutation may further contribute to the increase in affinity, assuming an additive effect of the two mutations. Given that B22‐1 and B22‐15 had essentially the same affinities (*K*_D_s 3.56 nM and 3.06 nM), the extra CDR2 H52N substitution in B22‐15 points to the possible neutral role of this mutation. If H52N is indeed affinity‐neutral, the substantial drop in affinity for B22‐14 (*K*_D_ 7257 nM vs *K*_D_ 295 nM for B22) with the double CDR2 H52N/Y58S mutations must be attributed to the Y58S mutation. This mutation is also present in the second affinity‐diminished V_H_, B22‐4; however, in this case, the beneficial H35Y mutation appears to mitigate the negative effect of Y58S, resulting in a smaller decrease in affinity (*K*_D_ 490 nM vs *K*_D_ 295 nM for B22). Our affinity maturation data indicate affinity improvements were achieved through various mutational combinations across different V_H_s: in B22‐1 and B22‐15, through CDR1 H35Y / CDR3 L100N mutations; while in B22‐2, B22‐5, and B22‐19, through CDR1 H35Y / CDR2 I51E, CDR1 H35Y / CDR2 V63T, and CDR1 H35Y / CDR2 A60T mutations, respectively. Notably, affinity improvements did not compromise PrA binding; unsurprising given the minimal number of mutations introduced into B22. Affinity‐matured V_H_s were capable of binding to spike protein expressed on the CHO cell surface, an arrangement that closely mimics spike protein presentation on the surface of SARS‐CoV‐2. Importantly, these V_H_s also neutralized the interaction of RBD with the ACE2 receptor. *IC*_50_s of 22.8 nM and 29.8 nM for B22‐15 and B22‐19 are comparable to those for some immune V_H_Hs (Rossotti, van Faassen, et al., 2022), suggesting that the optimization process presented here has the potential to yield efficacious neutralizing sdAbs.

However, the enhancement in affinity was associated with increased aggregation propensity. Relative to the B22 V_H_, the five affinity‐improved V_H_s were more aggregation‐prone, while the two affinity‐diminished V_H_s, B22‐4 and B22‐14, were less so. B22‐14, with a ~ 25‐fold drop in affinity compared to B22, was found to be aggregation‐resistant. These observations align with known trade‐offs between antibody affinity and other key properties such as specificity, stability, and solubility. They also support prior studies indicating that affinity maturation processes, whether occurring in vivo or conducted in vitro, can lead to antibody destabilization (Julian et al., 2015; Julian et al., 2017; Makowski et al., 2022; Rabia et al., 2018; Stimple et al., 2020; Sun et al., 2013, 2018; Tiller et al., 2017; Wang et al., 2013, 2017). While we did not conduct systematic mutational studies here, a preliminary hypothesis may connect the H35Y mutation to aggregation behavior. In support of this, all six aggregation‐prone mutants contained H35Y, while B22‐14, which was non‐aggregating, lacked this mutation. Consistent with this, previous evaluations of stability‐sequence diversity trade‐offs in the VHB82_SS_ library indicated that stability biases in the library favored Asp, His, Pro, and Gln while disfavoring aromatic residues Phe, Trp, and Tyr (Henry et al., 2017). Studies have also highlighted the role of position 35 in CDR1 concerning solubility in both V_H_s and V_H_Hs (Dudgeon et al., 2009; Jespers et al., 2004; Kim et al., 2014; Spinelli et al., 1996).

Regarding the affinity‐specificity trade‐offs, the affinity‐improved V_H_s did not bind to Fc and casein or to human serum albumin and ovalbumin—two reagents commonly used as polyspecificity reagents to assess the specificity of antibody immunotherapeutics (Makowski et al., 2021) —at concentrations as high as ~100 to 600 times the *EC*_50_/*K*_D_ values of the V_H_s. While these results indicate the absence of non‐specific interactions, more comprehensive and rigorous specificity binding studies using a wider range of polyspecificity reagents are needed to determine whether an affinity‐specificity trade‐off exists. The effects of mutations on expression yields varied, and potential affinity‐expression trade‐offs were not clearly observed.

In contrast to previous findings that noted affinity‐thermodynamic stability (i.e., thermostability) trade‐offs for antibodies including human V_H_s and camelid V_H_Hs (Julian et al., 2015; Makowski et al., 2022; Tiller et al., 2017), no such trade‐offs were observed here. All affinity‐improved V_H_s maintained equal or higher thermostability (*T*_m_) than the wild‐type B22 V_H_, while the monomeric, affinity‐diminished B22‐14 exhibited a lower *T*_m_. Furthermore, the mutation sets in B22‐1 and B22‐15, which were more beneficial for affinity than those in B22‐2, B22‐5, and B22‐19, also proved more advantageous for thermostability. Collectively, this suggests that while affinity‐improving mutations are closely linked to those improving thermostability, they are mutually exclusive with aggregation‐resistance mutations. Therefore, additional mutations in the domain—either in CDR or FR (e.g., camelization)—are needed to compensate for the loss of aggregation‐resistance in stability‐ and affinity‐improved V_H_s (Julian et al., 2017; Sun et al., 2013, 2018; Wang et al., 2013, 2017). Nonetheless, systematic reversion mutational analyses are necessary to determine the individual impacts of the mutations on affinity, thermostability, and aggregation resistance of the affinity‐improved variants. For instance, such an approach with human V_H_ and V_H_H variants has shown that affinity‐enhancing mutations can have positive, negative, or neutral effects on antibody thermostability and specificity (Julian et al., 2017; Tiller et al., 2017). Since the affinity‐improved V_H_s in this study exhibited the same or higher *T*_m_s than the wild‐type B22 V_H_, their increased tendency to aggregate likely results from decreased colloidal stability rather than reduced thermodynamic stability.

Several studies have showed camelization involving the introduction of 3–4 camelid V_H_H residues into human V_H_s at Kabat hallmark positions 37, 44, 45, and 47 can convert aggregating V_H_s into non‐aggregating ones (Davies & Riechmann, 1994, 1995, 1996a, 1996b; Dottorini et al., 2004; Kim et al., 2014; Tanha et al., 2001). Here we demonstrate that partial camelization, which involves introducing as few as 2 camelid V_H_H residues at positions 44 and 45, can convert an aggregating V_H_ (B22‐15) into a non‐aggregating variant (B22‐15‐2C) without compromising antigen binding affinity or PrA binding. While partial camelization minimally alters the humanness of the V_H_ sequence, it remains uncertain whether it also preserves its low immunogenicity potential, as the camelized substitutions are often solvent‐exposed and of foreign origin, potentially introducing a risk of immunogenicity (Fernandez‐Quintero et al., 2024). In contrast, full camelization partially solubilized B22‐15 but significantly reduced its antigen binding affinity. PrA binding data suggest that excessive conformational changes in the fully camelized B22‐15‐4C V_H_ may have disrupted binding interactions, leading to its substantial decrease in affinity for RBD.

While our findings with the B22‐15‐2C/B22‐15‐4C V_H_ pair suggest that partial camelization is an effective approach for rapidly optimizing therapeutic human V_H_s, the broader applicability of this strategy—and the potential benefits of full camelization in certain contexts—remain uncertain. The impact of modifying hallmark residues at FR2 positions 37, 44, 45, and 47 in sdAbs has been discussed recently (Fernandez‐Quintero et al., 2024). Altering residues at these positions, such as in V_H_s, may improve one binder but could have drastic effects on another, particularly as the G44E/L45R mutations significantly change such residue properties as size and charge. Furthermore, the camelization tetrad found ineffective in this study is also present in highly soluble camelid V_H_Hs, further supporting the idea that solubility effects are context‐dependent. While we tested only one specific tetrad, camelid species use various combinations at the four hallmark FR2 positions—adaptations that evolved to enhance paratope size, diversity, and stability of bioactive conformations (Fernandez‐Quintero et al., 2024; Wu et al., 2020). A more comprehensive approach could involve screening multiple tetrads to identify those that best solubilize target V_H_s. Identifying a suitable subset of solubilizing tetrads could help streamline the solubilization process for human V_H_s.

## 4 CONCLUSIONS

In summary, we have presented an effective, immunization‐independent optimization approach to quickly generate human V_H_s of high affinity, solubility, and stability through in vitro affinity maturation followed by minimal camelization. Although the SARS‐CoV‐2 spike protein was used as a model antigen, we anticipate that the same workflow can be applied to other targets, offering a reliable pipeline for discovering therapeutic V_H_s. Our study exemplifies the common affinity‐stability/solubility/specificity trade‐offs encountered in antibody development. Efforts to overcome such trade‐offs have included employing unique library design and selection strategies that in principle mimic the in vivo process of somatic hypermutation, screening a sufficient/high number of mutants post library selection, and performing reversion mutational analysis, as well as complementary computational and machine learning methods (Julian et al., 2015; Makowski et al., 2022; Stimple et al., 2020; Tiller et al., 2017). Integrating these strategies into our optimization method could further enhance its efficacy.

## 5 MATERIALS AND METHODS

### 5.1 Recombinant antigens, antibodies and reagents

Purified recombinant spike protein and fragments from SARS‐CoV‐2 Wuhan were described previously (Rossotti, van Faassen, et al., 2022). Bio‐RBD was purchased from ACROBiosystems (Cat#SPD‐C82E9). Full‐length spike protein (1273 amino acids), C‐terminally His_6_‐tagged RBD‐H6 (RBD amino acids 319–591), RBD‐Fc (RBD amino acids 319–591), and Fc were produced in‐house. RBD‐H6 was conjugated using the DyLight650 NHS Ester kit (Thermo Fisher Scientific, Cat#62266) and purified using Zeba spin desalting columns of 7 K MWCO (Thermo Fisher Scientific, Cat#89882) according to the manufacturer's instructions. The resulting protein (RBD‐DL650) concentration and the degree of conjugation (~2.5) were measured and calculated using nanodrop One (Thermo Fisher Scientific). Human serum albumin was obtained from Sigma (Cat#A8763) and dissolved in PBS at 1.5 mg/mL. Ovalbumin was obtained from Sigma (Cat#A5503) and dissolved in milliQ water at 1 mg/mL. Antibodies and other detection reagents used in this study included mouse monoclonal anti‐M13 major coat protein conjugated to horseradish peroxidase (anti‐M13 HRP) (Santa Cruz Biotechnology, Cat#Sc‐53,004), rabbit polyclonal anti‐6‐His Tag conjugated to HRP (anti‐His HRP) (Bethyl Laboratories, Cat#A190‐114P), Pierce™ Recombinant Protein A HRP conjugate (PrA HRP) (Thermo Fisher Scientific, Cat#32400), streptavidin conjugated to HRP (SA HRP) or to R‐Phycoerythrin (SA PE) (Thermo Fisher Scientific, Cat#21126 and Cat#S866, respectively), chicken polyclonal anti‐c‐Myc (anti‐c‐Myc) (Abcam, Cat#ab19233), goat anti‐chicken IgY (H + L) conjugated to DyLight650 (anti‐chicken IgY DL650) (Bethyl Laboratories, Cat#A30‐206D5), goat polyclonal anti‐chicken IgY (H + L) conjugated to Alexa Fluor 488 (anti‐chicken IgY AF488) (Abcam, Cat#ab150173), and goat anti‐human IgG Fc conjugated to HRP or fluorescein isothiocyanate (anti‐human Fc HRP and FITC) (Sigma, Cat#A0170 and Thermo Fisher Scientific, Cat#A18818, respectively). M13K07 helper phage (Cat#N0315S), Q5® Hot Start High Fidelity DNA Polymerase (Cat#M0493L) and dNTP mix (Cat#N0447L) were purchased from New England Biolabs. Sodium phosphate monobasic and dibasic were purchased from VWR (Cat#470302–666 and Cat#470302–660, respectively).

### 5.2 V_H_ phage display library selection and screening

The phage‐displayed fully human V_H_ synthetic library (VHB82_SS_) used in this study was previously described (Henry et al., 2017). Library phage particles preparation and panning experiments were carried out essentially as described (Baral et al., 2013). Briefly, for panning, different formats of antigens (strategy A, B, and C) were used in order to increase the likelihood of selecting sdAbs targeting a wide variety of epitopes on the RBD (Figure S1). Antigens were captured or passively adsorbed in appropriate wells overnight at 4°C with agitation. In strategy A, 0.5 μg/well of Bio‐RBD in 100 μL of phosphate‐buffer saline (PBS) was captured in streptavidin‐ or neutravidin‐coated wells already pre‐blocked with Superblock™ Buffer (Thermo Fisher Scientific, Cat#15501 and Cat#15127, respectively). The same conditions were used for rounds 1 and 3 of strategy B in which 10 μg/well of the full‐length spike protein in 100 μL of PBS was directly adsorbed in wells of NUNC® Immulon 4 HBX microtiter plates (Thermo Fisher Scientific, Cat#3855) at round 2. Finally, for strategy C, 5 μg/well of Fc or RBD‐Fc in 100 μL of PBS was applied to separate wells of NUNC® Immulon 4 HBX microtiter plates. For this strategy, the Fc was used for negative selection of input phages prior to incubation with the target antigen. For the other two strategies, negative selection was achieved on empty wells prior to incubation with the target antigen. The next morning, wells that were not pre‐blocked were emptied and 300 μL/well of blocking buffer (PBS containing either 1% [w/v] of casein [PBSC] or 5% [w/v] of skim milk [PBSS]) was added in each well for 1 h at room temperature prior to incubation with library phages (1 × 10^12^ colony‐forming units in blocking buffer containing 0.05% [v/v] Tween 20) for 2 h at room temperature. The blocking buffer (PBSC and PBSS) was switched in alternate rounds of panning, except for panning on streptavidin‐ and neutravidin‐coated wells which were already pre‐blocked. In the latter case, the capture protein (streptavidin and neutravidin) was switched in alternate rounds of panning. Following the phage incubation, wells were washed 10 times with PBS containing 0.05% (v/v) Tween 20 (PBST) and bound phage was eluted with 100 μL of 100 mM triethylamine for 10 min at room temperature, neutralized with 2 M Tris–HCl pH 8, and used to infect exponentially‐growing *E. coli* TG1 cells. The cultured *E. coli* were then superinfected with M13K07 helper phage at a multiplicity of infection of 20:1 at 37°C for 30 min with no shaking, after which they were grown overnight at 37°C and 250 rpm in fresh 2 × YT media in the presence of 100 μg/mL of ampicillin and 50 μg/mL of kanamycin. The next day, amplified phage was purified by polyethylene glycol precipitation from 10 mL overnight cultures and used in subsequent panning rounds. After three rounds of selection, V_H_‐displaying phage clones were screened for antigen binding by polyclonal phage ELISA as described below. Positive V_H_s were identified by DNA sequencing.

### 5.3 V_H_ cloning and expression in *E. coli*

For expression of V_H_s, selected sequences from panning were cloned into the SfiI sites of a modified pET expression vector containing biotin acceptor peptide and poly‐histidine tags (pMRO‐BAP‐H6; (Trempe et al., 2022)) for their production in BL21(DE3) *E. coli*. Affinity‐matured variants of B22 and the camelized variants of B22‐15 subcloned in the pMRO‐BAP‐H6 expression plasmid were synthesized by TWIST BioScience. Following electroporation of BL21(DE3) cells with the plasmids, individual colonies were cultured overnight in 10 mL of 2 × YT media supplemented with 50 μg/mL of kanamycin (2 × YT‐Kan) at 37°C and 250 rpm. The next day, cultures were added to 250 mL of 2 × YT‐Kan and grown to an A_600nm_ of 0.6. Expression of V_H_s was induced with 10 μM of isopropyl β‐D‐1‐thiogalactopyranoside (IPTG) overnight at 28°C and 250 rpm. Bacterial pellets were then harvested by centrifugation at 6129 × *g* for 15 min at 4°C, and V_H_s were extracted by sonication and purified by IMAC as previously described (Baral et al., 2013). When applicable, V_H_s were enzymatically biotinylated prior to their purification by IMAC by incubating 1 mg of purified V_H_s with 10 μM of ATP (Alfa Aesar, Cat#CAAAJ61125‐09), 100 μM of D‐biotin (VWR, Cat#97061–446) and bacterial cell extracts overexpressing *E. coli* biotin ligase (BirA) as previously described (Rossotti et al., 2015). Purified V_H_s were confirmed to be intact by mass spectrometry.

### 5.4 In vitro expression and biotinylation of V_H_s

In vitro expression of the wild‐type B22 and its affinity‐matured variants in the pMRO‐BAP‐H6 vector was achieved using the S30 T7 High‐Yield Protein Expression System (Promega, Cat#L1110) according to the manufacturer's recommendations. Expression reactions were performed in the presence of 1 mM of D‐biotin and 1 μg of *E. coli* BirA plasmid DNA to achieve simultaneous biotinylation of expressed proteins. V_H_s were buffer exchanged in PBS using Amicon® Ultra‐15 Centrifugal Filter Units (Millipore‐Sigma, Cat#UFC905024) prior to their binding assessment by ELISA (see below).

### 5.5 Size‐exclusion chromatography

Purified V_H_s were subjected to SEC to determine their aggregation tendencies and to isolate monomeric fractions. Prior to SEC analysis, V_H_s were maintained under consistent conditions regarding storage buffer, temperature, and duration. Briefly, except for B14 and C2‐57, where the protein expression yields were too low, 1 mg of each V_H_ (in 800 μL) was injected into a Superdex™ 75 10/300 GL column (Cytiva, Cat#29148721) connected to an ÄKTA FPLC protein purification system (Cytiva) as previously described (Baral et al., 2013). PBS was used as the running buffer at a flow rate of 0.5 mL/min for a total of 54 min per sample at room temperature. Fractions corresponding to the monomeric peak were collected, aliquoted, and stored at −80°C until further use. % monomers represent the fraction of the area under the monomeric peak over the total peak area and were calculated using the UNICORN™ 7.6 software (Cytiva).

### 5.6 Melting temperature measurements

*T*_m_s were determined by CD as previously described (Henry et al., 2017). Ellipticity of V_H_s at 100 μg/mL was measured at a wavelength of 202 nm in 100 mM sodium phosphate buffer, pH 7.4. Ellipticity measurements were normalized to a percentage scale, and then, *T*_m_s were determined by plotting % V_H_ folded vs temperature and fitting the Boltzmann sigmoidal (non‐linear regression) model to the plot using GraphPad Prism version 10.1.2 for Windows (GraphPad Software, www.graphpad.com).

### 5.7 ELISA

*(a) Polyclonal phage ELISA*. Wells of NUNC® Immulon 4 HBX microtiter plates were coated with 100–500 ng/well of each antigen in 100 μL of PBS overnight at 4°C with slow agitation. The next morning, wells were blocked with 300 μL of PBSC for 1 h at room temperature, followed by the addition of amplified phage products from each round of panning serially diluted in PBSC/0.05% (v/v) Tween 20 (PBSCT). Following incubation for 2 h at room temperature, wells were washed three times with PBST and once with PBS before adding 100 μL of PBST containing anti‐M13 HRP detecting antibody for 1 h at room temperature. Wells were then washed as above prior to incubation with 100 μL of TMB substrate (SeraCare, Cat#50–76‐00) for 5–30 min. The reaction was stopped by adding 100 μL of 1 M H_2_SO_4_, and A_450nm_ was read with a microtiter plate reader. *(b) ELISA with V*_*H*_ *‐containing bacterial lysates*. Eluted phage products from round 3 of panning were subcloned in the SfiI sites of the pMRO‐BAP‐H6 plasmid. Individual colonies were grown in 0.4 mL of 2 × YT‐Kan in a 96 deep well plate at 37°C and 250 rpm until an A_600nm_ of 0.5 was achieved. The expression of V_H_s was induced with 10 μM of IPTG at 28°C and 250 rpm overnight. The next day, bacterial pellets were harvested by centrifugation at 6129 × *g* for 15 min at 4°C, followed by the extraction of the V_H_s using B‐PER™ bacterial protein extraction reagent (Thermo Fisher Scientific, Cat#78248) as per the manufacturer's recommendations. Following lysis, cell lysates were cleared by centrifugation at 10,000 × *g* for 20 min at 4°C, and V_H_‐containing supernatants diluted 5‐fold in PBS were used for ELISA as described in part *(a)* using anti‐His HRP for detection. *(c) PrA ELISA*. Purified biotinylated V_H_s diluted to 200 nM in PBSCT were captured in streptavidin‐coated wells overnight at 4°C with slow agitation. The next morning, wells were washed as described in part *(a)*, followed by detection with anti‐His HRP or PrA HRP. *(d) Affinity screen by ELISA*. In vitro‐expressed samples containing biotinylated V_H_s in PBS were added to streptavidin‐coated wells and incubated at 4°C overnight with slow agitation. The next morning, wells were washed as described in part *(a)* followed by detection with anti‐His HRP or incubation with 100 μL of PBSCT containing RBD‐Fc at 1 μg/mL for 1 h at room temperature. Following incubation and washes as above, detection of bound RBD‐Fc was achieved using anti‐human Fc HRP. *(e) ELISA with purified V*_*H*_ *s*. Serially diluted biotinylated V_H_s in PBSCT were added to wells containing the antigens, and assays were carried out as described in part *(a)*. SA HRP was used to detect binding. *EC*_50_s for the binding of V_H_s to RBD‐Fc were determined by plotting A_450nm_ (binding) vs [sdAb] and fitting the specific binding with Hill slope (non‐linear regression) model to the plot using GraphPad Prism version 10.1.2 for Windows.

### 5.8 Surface plasmon resonance binding assays

All monomeric human V_H_s were purified by SEC into HBS‐EP buffer (10 mM HEPES, 150 mM NaCl, 3 mM ethylenediaminetetraacetic acid [EDTA], 0.005% [v/v] Tween 20, pH 7.4), or PBST buffer (135 mM NaCl, 2.7 mM KCl, 4.3 mM Na_2_HPO_4_, 1.4 mM KH_2_PO_4_, 3 mM EDTA, 0.05% [v/v] Tween 20, pH 7.2) prior to SPR analyses. All SPR analyses were performed on a Biacore™ T200 instrument (Cytiva) at 25°C. Briefly, proteins (RBD‐Fc, 725–869 resonance units [RUs]; Fc, 303–442 RUs; PrA, 650 RUs; ovalbumin, 510 RUs) were immobilized via amine coupling on CM5 series S sensor chips (Cytiva, Cat#29149603) in 10 mM acetate buffer, pH 4.0, according to the manufacturer's instructions. This immobilization densities resulted in surfaces with theoretical *R*_max_s ranging from 186–272 RUs. V_H_s were injected at various concentrations ranging from 2.5 nM to 8 μM, at a flow rate of 20 μL/min, with contact times ranging from 30 to 180 s and dissociation times from 120 to 300 s. All surfaces were regenerated using 10 mM glycine, pH 1.5, for 6 s at 100 μL/min (30 s at 100 μL/min for ovalbumin). Ethanolamine‐blocked flow cells served as reference surfaces. Single‐cycle kinetic data were fitted to a 1:1 interaction model and binding affinities (*K*_D_s) and kinetic rate constants (*k*_a_s, *k*_d_s) were determined using BIAevaluation 3.2 software (Cytiva).

### 5.9 Affinity maturation using yeast surface display

*(a) YSD V*_*H*_ *variants library construction*. Toward improving the affinity of B22, a YSD library of B22 variants of single‐site saturated mutagenesis targeting the three CDRs—10 CDR1 positions (amino acids 26–35), 17 CDR2 positions (amino acids 50–65) and 10 CDR3 positions (amino acids 93–102) was created. The variants were synthesized at IDT as an oligo pool with NNK at each targeted amino acid residue and with the FR codons optimized for expression in yeast *Saccharomyces cerevisiae*. The synthesized oligo pool was then amplified by polymerase chain reaction (PCR) with primers OCW2076, OCW2078R, OCW1453, and OCW1454R (Table S3) to make the full‐length V_H_ fragment with flanking sequences that overlap with the YSD vector pPNL6 (Feldhaus et al., 2003). The PCR product was then purified using QIAquick spin columns (Qiagen, Cat#28104) and cloned into smaI‐cut, gel‐purified pPNL6‐derived YSD vector through in vivo recombination in the yeast strain YCW2052 using the high‐efficiency lithium acetate transformation protocol (Gietz & Woods, 2002). The transformation was carried out in five reactions, each containing approximately 500 ng of vector DNA and 100 ng of amplified oligo pool DNA; the yeast cells of the reactions were then pooled and grown in selective media of synthetic dextrose tryptophan‐dropout media SC‐trp/Glu (1.92 g/L Trp‐dropout [US Biological Life Sciences, Cat#D9530], 6.7 g/L Yeast nitrogen base without amino acids [BD Difco, Cat#0919073], 20 g/L glucose [Sigma‐Aldrich, Cat#G8270]) at 30°C to saturation (~40 h). The cells were collected and stored in the selective media supplemented with 10% (v/v) glycerol in aliquots at −80°C. The number of primary transformation colonies of the yeast display library was estimated at 1.2 × 10^5^. *(b) YSD V*_*H*_ *library expression induction*. An overnight culture containing ~10^7^ of yeast cells of the YSD‐B22 construct in 10 mL of pre‐induction medium (SC‐trp/GluR+Pi+PS; 1.92 g/L Trp‐dropout, 6.7 g/L Yeast Nitrogen Base without amino acids, 20 g/L glucose, 10 g/L raffinose [Sigma‐Aldrich, Cat#R0250], 100 mM Na_2_HPO_4_, pH ~6.7), supplemented with 1x penicillin/streptomycin (Gibco, Cat#15149–122)) was grown overnight in a shaker incubator at 30°C and 225 rpm. To induce the expression of the yeast display of the B22 variants, approximately five A_600nm_ of yeast cells (~10^8^ cells) from the overnight culture were pelleted by centrifugation at ~3000 × *g* for 5 min at room temperature, resuspended in the SC‐trp/GalR+Pi+PS induction medium (pre‐induction medium but with galactose [Sigma‐Aldrich, Cat#G0750] instead of glucose, supplemented with 1x penicillin/streptomycin) at a cell density of ~5 × 10^6^ cells/mL and grown in a shaker incubator at 22°C and 225 rpm for 16–30 h. Yeast cells were collected by centrifugation and washed once in PBS in preparation for labeling with desired antigens. *(c)YSD V*_*H*_ *library labeling and selection*: ~10^8^ of induced yeast cells were blocked with PBS containing 2% (w/v) bovine serum albumin (BSA) and 0.05% (v/v) Tween 20, followed by incubation with 2 μg/mL of anti‐c‐Myc antibody diluted in PBS with 1% (w/v) BSA and 0.025% (v/v) Tween 20 at 4°C for 1 h. The cells were washed twice with PBS containing 0.05% (v/v) Tween 20 and collected by centrifugation at 3300 × *g* for 1 min at 4°C. The pelleted cells were then resuspended, stained with anti‐chicken IgY DL650 at 0.5 μg/mL for 45 min at 4°C, and protected from light during the incubation and subsequent handling with the fluorescent conjugates. The cells were then washed as above and resuspended in 10 mL of PBS + 1% (w/v) BSA and sorted using a BD FACS Aria™ II (BD Biosciences) with a 70‐μm nozzle to select c‐Myc‐expressing cells. ~2 × 10^7^ cells (20% population) were collected, titrated, propagated in SC‐trp/Glu medium supplemented with 1x penicillin/streptomycin, and stocked as B22Lib‐R0. For antigen binding selection, ~10^6^ of induced B22Lib‐R0 cells were blocked as above, followed by probing with SARS‐CoV‐2 spike RBD antigens. For the first round of FACS selection (R1), cells were incubated with RBD‐Fc at 20 nM together with anti‐c‐Myc for 1.5 h at 4°C. Following washes, pelleted cells were stained with anti‐human Fc FITC diluted to 1.5 μg/mL and anti‐chicken IgY DL650 at 0.5 μg/mL for 45 min at 4°C. The cells were then washed and resuspended in 4 mL PBS + 1% (w/v) BSA for sorting with a gating setup to collect the c‐Myc^+^ RBD^+^ double‐positive (FITC/DL650) population at ~10% of the input to generate B22Lib‐R1. Subsequent rounds of FACS selection (R2, R3, and R4) were performed sequentially with induced cells of the previous‐round selection (B22Lib‐R1 for R2; B22Lib‐R2 for R3, and B22Lib‐R3 for R4). Cells were first incubated with chicken anti‐c‐Myc for 1 h at 4°C, then washed and collected by centrifugation. Pelleted cells were then co‐stained with RBD‐DL650 at 200 nM and AF488‐conjugated anti‐chicken IgY at 0.25 μg/mL for 1.5 h at 4°C. Following incubation, cells were washed and sorted as described above with a gating setup to collect c‐Myc^+^ RBD^+^ double‐positive (AF488/DL650) population.

### 5.10 NGS analysis of yeast surface display libraries

*(a). DNA extraction and NGS library preparation*. DNA of B22Lib‐R0 and FACS‐selected B22Lib‐R3 were isolated from approximately 50 mg cell pellets using Yeast DNA extraction kit (Thermo Fisher Scientific, Cat#78870) according to the manufacturer's instructions. The genomic DNA was precipitated using isopropyl alcohol, washed with 70% ethanol to remove residual salts, dried and resuspended in 50 μL Tris‐EDTA buffer. To generate Illumina NGS libraries, a 2‐step PCR was performed. Six forward primers (KB‐Illumina‐1F/2F/3F/4F/5F/6F) were designed with a different number of spacer bases added downstream to the sequencing site to offset the order of sequencing and allow proper clustering. First PCR was done with each of the six forward primers combined with a single unique reverse primer (KB‐P7‐Universal‐1R); second PCR was carried out with universal primers (P5‐seqF, P7‐index1‐seqR, P7‐index2‐seqR) to introduce in adapters and indexes. The primers are listed in Table S3. For the first‐step PCR, two sets of six PCRs were performed each in a 50‐μL reaction mixture containing 650 ng of DNA template (R0 or R3), 0.5 μM of gene‐specific primers, 250 μM of dNTP mix and 0.02 U/μL of Q5® Hot Start High Fidelity DNA Polymerase. The PCR reactions were carried out with a ProFlex PCR system (Applied Biosystems, Life technologies) under the following conditions: initial denaturation of 1 min at 98°C followed by 21 cycles of 12 s at 98°C, 25 s at 70°C, and 15 s at 72°C, and a final extension step of 2 min at 72°C. PCR products from the same DNA template were pooled and purified using QIAquick PCR purification column (Qiagen) according to the manufacturer's instructions. The second‐step PCRs were performed in 50 μL of reaction mixture containing 1 ng of DNA template from the first‐step PCR, 0.5 μM of primers (P5‐seqF with P7‐index1‐seqR or with P7‐index2‐seqR), 250 μM of dNTP mix, and 0.02 U/μL of Q5® Hot Start High Fidelity DNA Polymerase in a ProFlex PCR system under the following conditions: initial denaturation of 30 s at 98°C followed by 13 cycles of 10 s at 98°C, 20 s at 60°C, and 20 s at 72°C, and a final extension step of 2 min at 72°C. PCR products were purified with QIAquick PCR purification column followed by a second purification using SPRI select magnetic beads (Beckman Coulter, Cat#B23318). Quantification and purity assessment of NGS libraries were done by quantitative PCR (qPCR) using NEBNextLibrary Quant kit according to the manufacturer's instructions (New England Biolabs, Cat#E7630). The samples were diluted to 4 nM for sequencing on an Illumina MiSeq using 2 × 300 bases paired end V3 kit (Illumina) at a mean depth of 288,576 reads. *b) NGS data analysis*. Sequencing data was aligned with STAR‐2.7.1a against the reference sequence. The output was filtered with an in‐house R script to keep only the uniquely mapped reads with a maximum of 10 mismatches; for the region where the two reads overlap, the base with the higher Phred score was kept. Bases with Phred score lower than Q30 were set to 30 if they were similar to the template, and remaining bases with Phred score lower than Q30 were set to “N”. The reads were then translated to amino acid sequence, ambiguous amino acids due to the presence of an “N” at a key position of a codon appeared as an “X”. Only sequences with one mutation were kept. The abundance of each amino acid type at each position was calculated and corrected across samples for sequencing depth by dividing the value by the total number of mapped reads and multiplying by 10^5^. Then, the fold‐change ratio was calculated using the formula log2((R3 + 1)/(R0 + 1)) and plotted as a heatmap using the pheatmap R package (https://cran.r-project.org/web/packages/pheatmap/index.html).

### 5.11 Molecular modeling

V_H_ structure modeling was originally done with ABodyBuilder (Leem et al., 2016; Leem & Deane, 2019; Leem et al., 2018) using default parameters. Retrospective models were also generated with a more recent version of the software, NanoBodyBuilder2 (Abanades et al., 2023). PyMol 2.4.0 (Schroedinger, LLC) was used for structural visualization, manipulation, and calculation of physico‐chemical properties, including inter‐atomic distances and molecular surface areas. While models generated by different tools exhibited certain differences mainly in the CDR3 loop, they were considered equivalent for the accuracy level required for mutant selection in this study. Solubility predictions were carried out with the structurally corrected CamSol method (Sormanni et al., 2015, 2017) using 3D structures predicted with the NanoBodyBuilder2 method (Abanades et al., 2023).

### 5.12 Cell binding assay

CHO^55E1^™ cells expressing the full‐length (including transmembrane and C‐terminal domains) SARS‐CoV‐2 Wuhan spike protein (CHO‐Spike) under the control of the cumate‐inducible CR5 promoter were generated by methionine sulfoximine selection of plasmid‐transfected cells, as described previously (Poulain et al., 2017). Non‐transfected CHO^55E1^™ cells (Parental CHO) were used as a negative antigen control. Cells were grown in BalanCD™ CHO Growth A medium (Irvine Scientific, Cat#91128) supplemented with 50 μM methionine sulfoximine at 120 rpm and 37°C in a humidified 5% CO_2_ atmosphere. Expression of the spike protein was induced by adding cumate at 2 μg/mL for 48 h at 37°C. For cell cytometry experiments, induced cells were harvested by centrifugation and resuspended at 1 × 10^6^ cells/mL in PBS with 1% (w/v) BSA (PBSB). Cells were kept on ice until use. Starting at 400 nM, four‐fold dilutions of V_H_s as well as A20.1 V_H_H negative antibody control (Hussack et al., 2011) were prepared in V‐Bottom 96‐well microtiter plates (Globe Scientific, Cat#120130) and mixed with 50 μL of CHO‐Spike cells. Plates were incubated for 2 h on ice, washed three times with PBS by centrifugation for 2 min at 200 × *g*, and, then, incubated for an additional hour with 100 μL of SA PE (Thermo Fisher Scientific, Cat#S866) at 2 μg/mL diluted in PBSB. Then, cells were washed as above and resuspended in 100 μL of 500 nM Draq 5 (Abcam, Cat#ab108410‐1002) and transferred to a 384‐well microplate (Corning 3764, Fisher Scientific Cat#07–201‐013) for data acquisition on a Mirrorball® instrument (SPT Labtech) and analysis with the Cellista software (SPT Labtech). *EC*_50_s for the binding of V_H_s to CHO‐Spike cells were obtained by plotting the peak fluorescence intensity per cell (peak intensity/cell) vs [V_H_] and fitting the specific binding with a Hill slope (non‐linear regression) model to the plot using the GraphPad Prism version 10.1.2 for Windows.

### 5.13 Surrogate virus neutralization assay

The ability of V_H_s to inhibit the interaction of recombinant RBD‐Fc with the ACE2 receptor expressed at the cell surface was determined by combining 100 ng of RBD‐Fc with 1 × 10^5^ HEK293T ACE2 cells (BEI Resources, Cat#NR‐52511) in the presence of 5‐fold dilutions of V_H_s starting at a concentration of 1000 nM in a final volume of 150 μL of PBSB. In control assays, V_H_s were omitted (No sdAb) or replaced with negative antibody control A20.1 V_H_H (Hussack et al., 2011). Following incubation on ice for 1 h, cells were washed twice with PBSB by centrifugation for 5 min at 200 × *g* and then incubated for an additional hour on ice with 50 μL of anti‐human Fc FITC diluted to 250 ng/mL in PBSB. Cells were subsequently washed as above and resuspended in 100 μL of fresh PBSB for data acquisition on a Mirrorball® instrument (SPT Labtech) and analysis with the Cellista software (SPT Labtech). *IC*_50_s for the neutralization of RBD/ACE2 binding by the V_H_s were obtained by plotting MMFI (median mean fluorescence intensity) vs [sdAb] and fitting the non‐linear regression [inhibitor] vs response—variable slope (four parameters) model to the plot using the GraphPad Prism version 10.1.2 for Windows.

## AUTHOR CONTRIBUTIONS

**Kasandra Bélanger:** Conceptualization; data curation; formal analysis; investigation; methodology; validation; visualization; writing – original draft. **Cunle Wu:** Conceptualization; data curation; formal analysis; investigation; methodology; validation; visualization; writing – review and editing. **Traian Sulea:** Conceptualization; data curation; formal analysis; investigation; methodology; validation; visualization; writing – review and editing. **Henk van Faassen:** Investigation. **Deborah Callaghan:** Investigation. **Annie Aubry:** Investigation. **Marc Sasseville:** Investigation. **Greg Hussack:** Data curation; formal analysis; investigation; validation; visualization; writing – review and editing. **Jamshid Tanha:** Conceptualization; data curation; funding acquisition; investigation; methodology; project administration; resources; supervision; validation; visualization; writing – original draft; writing – review and editing; formal analysis.

## CONFLICT OF INTEREST STATEMENT

The authors declare no conflicts of interest.

## Supporting information


Data S1.

## Data Availability

All data are available upon request to Jamshid Tanha, the corresponding author.
