# Supplementary material for: Optimization of synthetic human VH affinity and solubility through in vitro affinity maturation and minimal camelization
Source: Protein Sci. 2025 Apr 22;34(5):e70114. doi: 10.1002/pro.70114 (PMC12012759; doi:10.1002/pro.70114)
Supplement: Supplementary file 1 — Data S1. [file PRO-34-e70114-s001.docx]

**List of Supplementary Materials**

Tables S1 – S3

Supplementary Figures S1 – S9

**Table S1. Kinetic (*k*_a_, *k*_d_) and equilibrium dissociation (*K*_D_) constants for the binding of B22 V_H_ variants to RBD-Fc.**

| **V_H_** | ***k*_a_ (M^-1^s^-1^)** | ***k*_d_ (s^-1^)** | ***K*_D_ (nM)** |
| --- | --- | --- | --- |
| B22 | (1.30 ± 0.01) × 10^5^ | (3.84 ± 0.03) × 10^-2^ | 295 ± 1 |
| B22-1 | (2.43 ± 0.01) × 10^5^ | (8.68 ± 0.03) × 10^-4^ | 3.56 ± 0.03 |
| B22-2 | (2.22 ± 0.00) × 10^5^ | (1.84 ± 0.00) × 10^-3^ | 8.30 ± 0.04 |
| B22-4 | (1.42 ± 0.01) × 10^5^ | (6.69 ± 0.01) × 10^-2^ | 490 ± 4 |
| B22-5 | (2.70 ± 0.00) × 10^5^ | (2.06 ± 0.00) × 10^-3^ | 7.61 ± 0.01 |
| B22-14 | - | - | 7257 ± 475 |
| B22-15 | (2.77 ± 0.02) × 10^5^ | (8.53 ± 0.02) × 10^-4^ | 3.08 ± 0.03 |
| B22-19 | (2.65 ± 0.01) × 10^5^ | (2.25 ± 0.00) × 10^-3^ | 8.47 ± 0.02 |

Values are mean ± SD of three technical replicates; B22-14 *K*_D_ was determined using a steady state model; The highest concentration of B22-14 flowed was below the estimated *K*_D_*.* *K*_D_s are recorded in Table 3. RBD-Fc, human IgG1 hinge/Fc-fused RBD.

**Table S2. Kinetic (*k*_a_, *k*_d_) and equilibrium dissociation (*K*_D_) constants for the binding of B22-15 V_H_ variants to RBD-Fc.**

| **V_H_** | ***k*_a_ (M^-1^s^-1^)** | ***k*_d_ (s^-1^)** | ***K*_D_ (nM)** |
| --- | --- | --- | --- |
| B22-15 | (3.18 ± 0.02) × 10^5^ | (1.89 ± 0.01) × 10^-3^ | 5.96 ± 0.04 |
| B22-15-2C | (2.86 ± 0.02) × 10^5^ | (1.86 ± 0.01) × 10^-3^ | 6.49 ± 0.05 |
| B22-15-4C | - | - | 3880 ± 100 |

Values are mean ± SD of three technical replicates; B22-15-4C *K*_D_ was determined using a steady state model; The highest concentration of B22-15-4C flowed was below the estimated *K*_D_*.* *K*_D_s are recorded in Table 4. RBD-Fc, human IgG1 hinge/Fc-fused RBD.

**Table S3. Primers used for NGS analysis.**

| **Prime**r | **Sequence (5’ 🡪 3’)** |  |
| --- | --- | --- |
| **PCR/IVR** |  | |
| OCW2076 | GCAGTGCTAGCGCCGCGGGTcaggtgcagctgcaggagtcggggggaggcttggtacagcctggggggtccctgagactctcctgt | |
| OCW2078R | CCTCCTCCTTGTCGACTGGCtgaagagacggtgaccgtggtacctttgcccca | |
| OCW1453 | GGCTAGTGGTGGAGGCGGTTCTGGCGGTGGAGGTAGCGGTGGCGGAGGCAGTGCTAGCGCCGCGGGT | |
| OCW1454R | GAGCTATTACAAGTCTTCTTCAGAAATAAGCTTTTGTTCGGATCCGCCCCCTCCAGATCCTCCTCCTCCTTGTCGACTGGC | |
| **NGS** |  | |
| KB-Illumina-1F | CGCTCTTCCGATCTCTGTCCCTGAGACTCTCCTGTGCA | |
| KB-Illumina-2F | CGCTCTTCCGATCTCTGAGATCCCTGAGACTCTCCTGTGCA | |
| KB-Illumina-3F | CGCTCTTCCGATCTCTGTCTAGATCCCTGAGACTCTCCTGTGCA | |
| KB-Illumina-4F | CGCTCTTCCGATCTCTGATGAATGGTTCCCTGAGACTCTCCTGTGCA | |
| KB-Illumina-5F | CGCTCTTCCGATCTCTGGATGCACATCTTCCCTGAGACTCTCCTGTGCA | |
| KB-Illumina-6F | CGCTCTTCCGATCTCTGCGATTGCTCGACTCCCTGAGACTCTCCTGTGCA | |
| KB-P7-Universal-1R | GTGCTCTTCCGATCTGACTGAAGAGACGGTGACCGTGGTACCTTT | |
| P5-seqF | AATGATACGGCGACCACCGAGATCTACACTCTTTCCCTACACGACGCTCTTCCGATCTCTG | |
| P7-index1-seqR | CAAGCAGAAGACGGCATACGAGATCGTGATGTGACTGGAGTTCAGACGTGTGCTCTTCCGATCTGAC | |
| P7-index2-seqR | CAAGCAGAAGACGGCATACGAGATACATCGGTGACTGGAGTTCAGACGTGTGCTCTTCCGATCTGAC | |

PCR, polymerase chain reaction; IVR, *in vitro* recombination; NGS, next-generation sequencing.


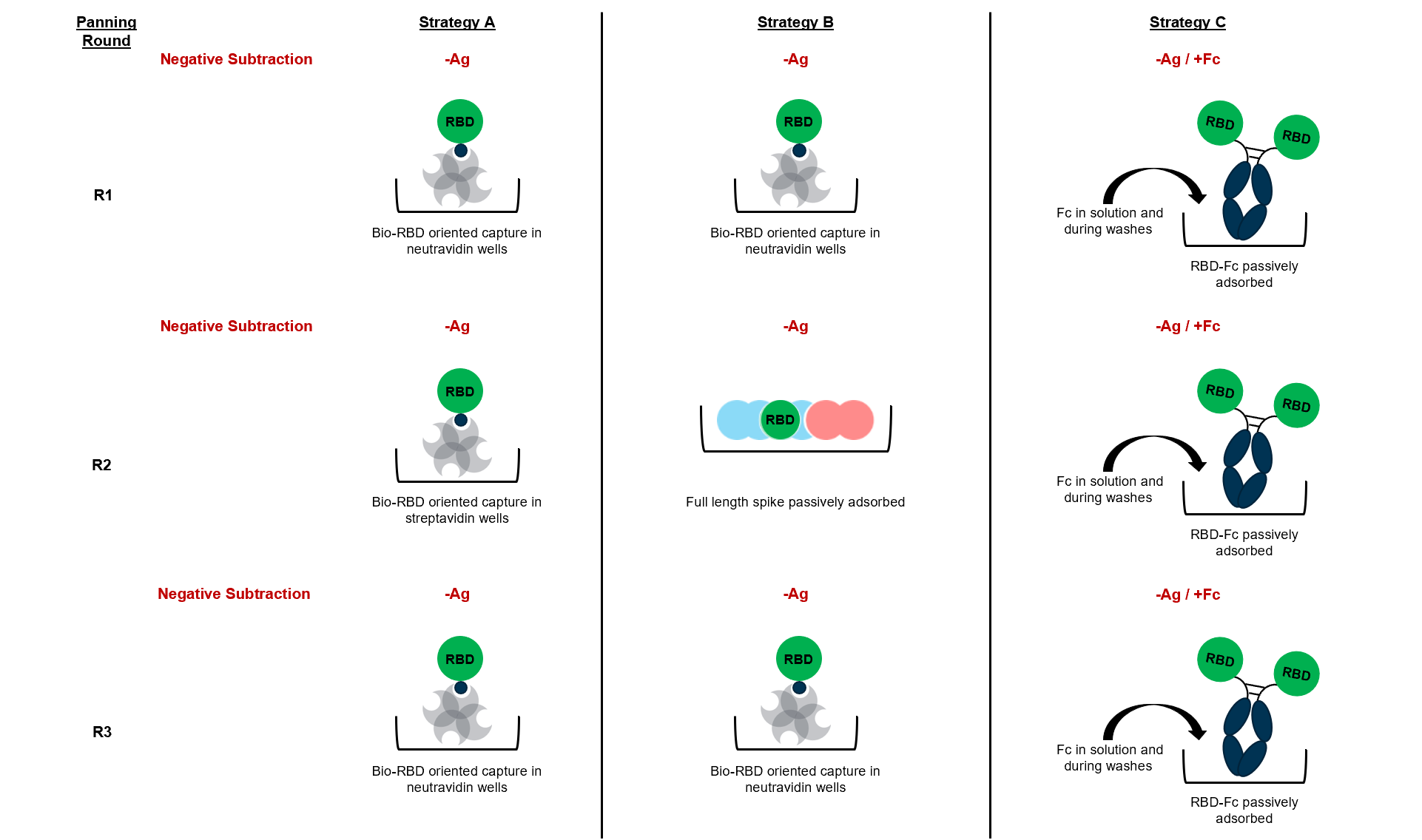


**Figure S1. Schematic representation of the three panning strategies employed to select human V_H_s against the RBD of SARS-CoV-2 spike protein.** Subtraction of the phage population on non-antigen-coated, blocked wells (“-Ag”; strategy A, B and C) and Fc control-coated, blocked wells (“-Ag / +Fc”; strategy C) was performed before incubation with the target antigen to eliminate non-specific and/or Fc-specific binders. For strategy C, the control Fc was also added in solution while incubating phage with the target antigen and during washes to increase the likelihood of eliminating Fc binders. In all three strategies, panning was conducted for three rounds. Bio-RBD, biotinylated RBD; Fc, human IgG1 hinge/Fc; RBD-Fc, human IgG1 hinge/Fc-fused RBD.

**Figure S2. Screening of human V_H_s for binding to SARS-CoV-2 spike protein RBD. a**) Following panning, serially diluted amplified polyclonal phage products from each of the three rounds of panning were added to the wells of an ELISA plate containing SARS-CoV-2 spike protein antigens (RBD-Fc, spike protein, RBD-H6, Bio-S1) and negative antigen controls (Fc, casein, streptavidin). Phage binding was then detected using an anti-M13 antibody conjugated to horseradish peroxidase (HRP). The pre-panned library was used as the negative control. Bio-S1, biotinylated S1 subunit of SARS-CoV-2 spike protein; RBD-H6, C-terminally His_6_-tagged RBD; Fc, human IgG1 hinge/Fc; RBD-Fc, human IgG1 hinge/Fc-fused RBD. **b**) Individual clones from round 3 of panning were expressed in bacteria at small scales followed by an extraction step using B-PER^TM^ bacterial protein extraction reagent. V_H_-containing cleared lysates were then tested by ELISA for binding to RBD-Fc antigen and negative antigen controls (Fc and casein) followed by detection using HRP-conjugated anti-His antibody. The two red and black circles represent the negative A20.1 ^1^ and positive VHH-72 ^2,3^ V_H_H antibody controls, respectively. V_H_s that demonstrated 1.5-fold higher binding signal than the A20.1 control against RBD-Fc and 1.5-fold increased binding signal against RBD-Fc compared to Fc (18 in total) were selected for further analysis.

**Figure S3. Sequences of (a) the 18 V_H_s isolated from the VHB82_SS_ human synthetic library following panning against RBD antigens and (b) the affinity-matured variants of the lead binder V_H_ B22.** The Kabat system of amino acid numbering scheme and FR/CDR designations are used ^4^. Mutated residues are shown in red. FR, framework region; CDR, complementarity-determining region.

**Figure S4. SPR sensorgrams illustrating the binding patterns of 17 V_H_s isolated after panning the VHB82_SS_ library against RBD-Fc and Fc.** Monomeric V_H_s were flowed at various concentration ranges over immobilized RBD-Fc and Fc, as described in MATERIALS AND METHODS. Binding kinetics and equilibrium dissociation constants (*k*_a_, *k*_d_ and *K*_D_) were determined through single-cycle kinetic analysis or steady-state affinity, using BIAevaluation 3.2 software, with results shown in Table 1. Black lines represent the raw data points, and red lines show 1:1 binding model fits. SPR assays were performed in triplicate, with results from one replicate shown. RU, resonance units.

**Figure S5. FACS selection of YSD B22 site saturation mutagenesis library.** Yeast cells of the unselected B22Lib (round 0, R0) were doubly stained with RBD-Fc (20 nM) and anti-c-Myc antibody followed by FITC- and DL650-conjugated secondary antibodies, and subjected to FACS to collect the gated FITC/DL650 doubly positive population (R1). Subsequent rounds of FACS selection (R2-R4) were performed with induced yeast cells of the previous round of selection (starting from R1) doubly strained with DL650-conjugated RBD (RBD-DL650, 200 nM) and anti-c-Myc antibody, along with AF488-conjugated secondary antibody to collect the gated doubly positive populations as indicated (upper panel). Yeast surface-displayed parental B22 (‘Parent’) was processed in parallel in each round to monitor the selection progress (lower panels). FACS, fluorescence-activated cell sorting; RBD-Fc, human IgG1 hinge/Fc-fused RBD.

**Figure S6. Relative mutational effects of CDR positions in antigen binding.** Heatmap of NGS analysis of round 3-selected (B22Lib-R3) *vs* unselected (B22Lib-R0) yeast cells of B22 library was plotted as Log_2_ enrichment ratios from the R3 *vs* R0 for substitutions that were mostly enriched (dark red) or depleted (dark blue, gradient scale is shown on the right side of the figure). Wild-type amino acids are in black squares. The CDR sequence of wild-type B22 V_H_ targeted for site-saturated mutagenesis is shown horizontally, and amino acid substitutions are shown vertically. Asterisk (*) denotes stop codon. The Kabat/IMGT (CDR1), Kabat (CDR2) and IMGT (CDR3) systems of CDR designations are used ^4,5^. The amino acid numbering scheme is according to the Kabat system ^4^. CDR, complementarity-determining region.


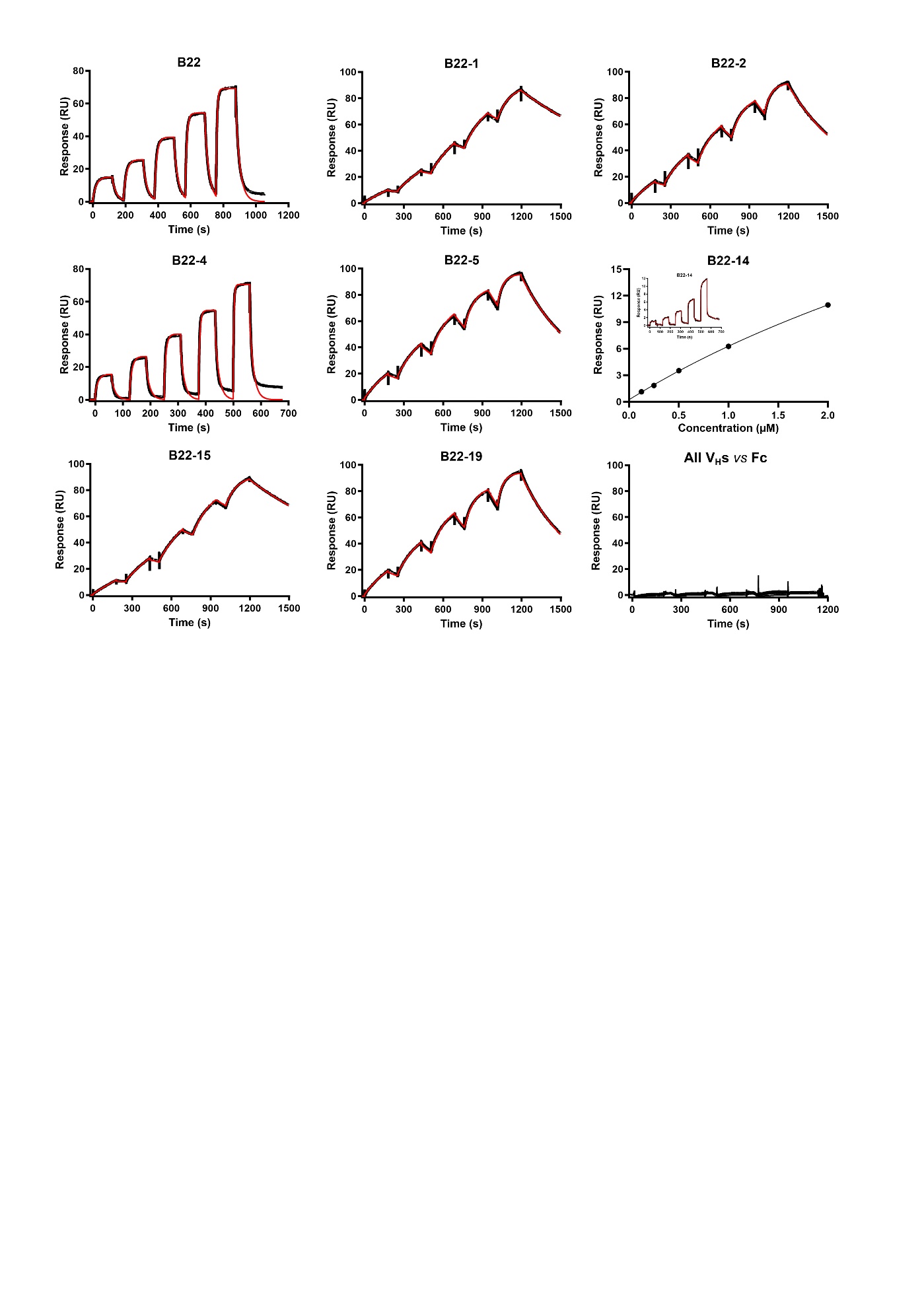


**Figure S7. SPR sensorgrams illustrating the binding of affinity-improved B22 V_H_s to RBD-Fc (first eight panels) and Fc (last panel).** Monomeric V_H_s were flowed at the following concentration ranges over immobilized RBD-Fc: B22 (62.5 – 1000 nM); B22-1 and B22-15 (2.5 – 40 nM); B22-2, B22-5 and B22-19 (5 – 80 nM); B22-4 and B22-14 (125 – 2000 nM). For flowing over Fc, each V_H_ was injected at 8, 40, 200 and 1000 nM. Binding kinetics and equilibrium dissociation constants (*k*_a_, *k*_d_ and *K*_D_) were determined through single-cycle kinetic analysis or steady-state affinity, using BIAevaluation 3.2 software, with results shown in Table 3 and Table S1. Black lines represent the raw data points, while red lines show 1:1 binding model fits. SPR assays were performed in triplicate, with results from one replicate shown. RU, resonance units.

**Figure S8. SPR sensorgrams illustrating the binding profiles of the affinity-improved and camelized B22 V_H_s to ovalbumin.** A dilution series of monomeric V_H_s were each flowed at 8 nM - 1 µM over immobilized ovalbumin. None of the V_H_s showed significant binding to ovalbumin (the theoretical maximum binding response of the ovalbumin surface was 190 RUs). V_H_s included in the assay are B22, B22-1, B22-2, B22-4, B22-5, B22-14, B22-15, B22-19, B22-15-2C and B22-15-4C. RU, resonance unit.

**Figure S9. Predicted *versus* measured solubilities of B22-15 and its camelized variants B22-15-2C and B22-15-4C.** A low-solubility V_H_, B42 (this study), and a high-solubility V_H_H antibody, 04 ^6^, which have CDR3 loops of similar lengths to B22 variants, are included as controls. Solubility scores are derived *in silico* with the structurally corrected CamSol method ^7,8^ and webserver (<https://www-cohsoftware.ch.cam.ac.uk/index.php/camsolstrucorr>) using 3D structures predicted with the NanoBodyBuilder2 method ^9^ and webserver (<https://opig.stats.ox.ac.uk/webapps/sabdab-sabpred/sabpred/nanobodybuilder2/>). Dotted line represents linear correlation through the data points shown. A very strong correlation between % monomer and solubility score is observed (r = 0.9799; p = 0.0034). CDR, complementarity-determining region 3.

**References**

1. Hussack G, Arbabi-Ghahroudi M, van Faassen H, Songer JG, Ng KK, MacKenzie R, Tanha J. Neutralization of *Clostridium difficile* toxin A with single-domain antibodies targeting the cell receptor binding domain. J Biol Chem. 2011; 286:8961-8976. doi:10.1074/jbc.M110.198754. PMID: 21216961.

2. Wrapp D, De Vlieger D, Corbett KS, Torres GM, Wang N, Van Breedam W, Roose K, van Schie L, COVID-19 Response Team, Hoffmann M, et al. Structural basis for potent neutralization of betacoronaviruses by single-domain camelid antibodies. Cell. 2020; 181:1004-1015 e1015. doi:10.1016/j.cell.2020.04.031. PMID: 32375025.

3. Wrapp D, De Vlieger D, Corbett KS, Torres GM, Wang N, Van Breedam W, Roose K, van Schie L, COVID-19 Response Team, Hoffmann M, et al. Erratum of "Structural basis for potent neutralization of betacoronaviruses by single-domain camelid antibodies". Cell. 2020; 181:1436-1441. doi:10.1016/j.cell.2020.05.047. PMID: 32531248.

4. Sequences of proteins of immunological interest. In: Kabat EA Sequences of proteins of immunological interest. Bethesda, MD :: U.S. Dept. of Health and Human Services, Public Health Service, National Institutes of Health; 1991.

5. Lefranc MP, Pommie C, Ruiz M, Giudicelli V, Foulquier E, Truong L, Thouvenin-Contet V, Lefranc G. IMGT unique numbering for immunoglobulin and T cell receptor variable domains and Ig superfamily V-like domains. Dev Comp Immunol. 2003; 27:55-77. doi:10.1016/s0145-305x(02)00039-3. PMID: 12477501.

6. Rossotti MA, van Faassen H, Tran AT, Sheff J, Sandhu JK, Duque D, Hewitt M, Wen X, Bavananthasivam J, Beitari S, et al. Arsenal of nanobodies shows broad-spectrum neutralization against SARS-CoV-2 variants of concern *in vitro* and *in vivo* in hamster models. Commun Biol. 2022; 5:933. doi:10.1038/s42003-022-03866-z. PMID: 36085335.

7. Sormanni P, Aprile FA, Vendruscolo M. The CamSol method of rational design of protein mutants with enhanced solubility. J Mol Biol. 2015; 427:478-490. doi:10.1016/j.jmb.2014.09.026. PMID: 25451785.

8. Sormanni P, Amery L, Ekizoglou S, Vendruscolo M, Popovic B. Rapid and accurate *in silico* solubility screening of a monoclonal antibody library. Sci Rep. 2017; 7:8200. doi:10.1038/s41598-017-07800-w. PMID: 28811609.

9. Abanades B, Wong WK, Boyles F, Georges G, Bujotzek A, Deane CM. ImmuneBuilder: deep-learning models for predicting the structures of immune proteins. Commun Biol. 2023; 6:575. doi:10.1038/s42003-023-04927-7. PMID: 37248282.
